# PET imaging and quantification of small animals using a clinical SiPM-based camera

**DOI:** 10.1186/s40658-023-00583-2

**Published:** 2023-10-07

**Authors:** Cédric Desmonts, Charline Lasnon, Cyril Jaudet, Nicolas Aide

**Affiliations:** 1 Nuclear Medicine Department, University Hospital of Caen, Avenue de La Côte de Nacre, 14033 Caen Cedex 9, France; 2 Normandy University, UNICAEN, INSERM 1086 ANTICIPE, Caen, France; 3 Nuclear Medicine Department, UNICANCER, Comprehensive Cancer Centre F. Baclesse, Caen, France; 4 Radiophysics Department, UNICANCER, Comprehensive Cancer Centre F. Baclesse, Caen, France

**Keywords:** SiPM PET, Preclinical research, Small-animal imaging, PET quantification

## Abstract

**Background:**

Small-animal PET imaging is an important tool in preclinical oncology. This study evaluated the ability of a clinical SiPM-PET camera to image several rats simultaneously and to perform quantification data analysis.

**Methods:**

Intrinsic spatial resolution was measured using 18F line sources, and image quality was assessed using a NEMA NU 4-2018 phantom. Quantification was evaluated using a fillable micro-hollow sphere phantom containing 4 spheres of different sizes (ranging from 3.95 to 7.86 mm). Recovery coefficients were computed for the maximum (Amax) and the mean (A50) pixel values measured on a 50% isocontour drawn on each sphere. Measurements were performed first with the phantom placed in the centre of the field of view and then in the off-centre position with the presence of three scattering sources to simulate the acquisition of four animals simultaneously. Quantification accuracy was finally validated using four 3D-printed phantoms mimicking rats with four subcutaneous tumours each. All experiments were performed for both 18F and 68Ga radionuclides.

**Results:**

Radial spatial resolutions measured using the PSF reconstruction algorithm were 1.80 mm and 1.78 mm for centred and off-centred acquisitions, respectively. Spill-overs in air and water and uniformity computed with the NEMA phantom centred in the FOV were 0.05, 0.1 and 5.55% for 18F and 0.08, 0.12 and 2.81% for 68Ga, respectively. Recovery coefficients calculated with the 18F-filled micro-hollow sphere phantom for each sphere varied from 0.51 to 1.43 for Amax and from 0.40 to 1.01 for A50. These values decreased from 0.28 to 0.92 for Amax and from 0.22 to 0.66 for A50 for 68 Ga acquisition. The results were not significantly different when imaging phantoms in the off-centre position with 3 scattering sources. Measurements performed with the four 3D-printed phantoms showed a good correlation between theoretical and measured activity in simulated tumours, with r^2^ values of 0.99 and 0.97 obtained for 18F and 68Ga, respectively.

**Conclusion:**

We found that the clinical SiPM-based PET system was close to that obtained with a dedicated small-animal PET device. This study showed the ability of such a system to image four rats simultaneously and to perform quantification analysis for radionuclides commonly used in oncology.

**Supplementary Information:**

The online version contains supplementary material available at 10.1186/s40658-023-00583-2.

## Introduction

Small-animal imaging is an important tool for diagnostic and therapeutic drug development. In vivo molecular imaging using positron emission tomography (PET) has become a technique of reference in preclinical research. Dedicated small-animal PET systems (SA-PET) have been developed specifically for this purpose. These devices have a high level of performance but are not widely available considering their high acquisition cost. To overcome this lack of accessibility, some authors have investigated the use of clinical PET systems for small-animal imaging [1–6]. Indeed, technological advancements introduced in recent decades, such as 3D-PET, time of flight (TOF) measurement or reconstruction algorithm modelling point spread function (PSF), have significantly enhanced the intrinsic performance of clinical PET systems [7]. These updates, both in hardware and software, have notably improved sensitivity, signal-to-noise ratio and spatial resolution, which are crucial factors for small-animal imaging. This type of equipment has the advantage of being available in all nuclear medicine facilities, and its use does not require additional investments for departments occasionally engaged in preclinical research. Clinical PET systems also have a much larger field of view than dedicated SA-PET cameras, which allows imaging several animals in the same acquisition. Indeed, with the development of molecularly targeted therapies and drug combinations requiring evaluation of different schedules, the number of animals to be imaged within a PET experiment can be large. Although the 3Rs rule has made it possible to significantly reduce the use of rodents in preclinical research [8], the number of animals needed to develop new therapeutics remains significant. High-throughput studies can be performed in high-resolution clinical PET systems by imaging more than one animal simultaneously. It has been reported in a previous study that it was possible to image four animals simultaneously on an analogic PET clinical system [9]. Most recently, a new generation of digital PET cameras has appeared. These systems, initially developed for PET/MR imaging and then made available for PET/CT devices, are based on the use of silicon photomultipliers (SiPMs) in place of conventional photomultiplier tubes (PMTs). The intrinsic performance and lesion detectability of these PET/CT systems have further increased compared to analogic cameras [10–13], which potentially increases their ability to image small animals.

In addition to the characterization of the tumour uptake through simple visual analysis, an important challenge in preclinical imaging is the ability of PET devices to perform in vivo quantification [14–16]. Indeed, many parameters related to the intrinsic performance of the camera and performance of the reconstruction algorithms, such as spatial resolution, sensitivity, attenuation and scattering correction, can create quantitative bias. It is thus important to evaluate the capacity of an imaging system to provide accurate quantitative measurements.

The aim of this study was to evaluate the feasibility of employing a clinical SiPM-based PET camera for small-animal imaging in preclinical research, as well as to evaluate its capability for performing image quantification analysis. The tests were conducted under conditions that closely resembled real preclinical examinations, utilizing 18F and 68Ga, which are two radionuclides widely used in preclinical research. First, the spatial resolution of the camera was estimated using 18F line sources, and image quality assessed using a dedicated SA-PET quality phantom. Then, quantification accuracy was estimated using a rat-sized phantom containing fillable microspheres and validated under preclinical conditions using a rat 3D-printed phantom.

## Material and methods

### PET camera

To implement this study, a Biograph VISION 450 camera was used (Siemens Healthineers, Knoxville, Tennessee). This camera consists of 6 rings of detectors with a total axial field of view coverage of 19.7 cm. Each detector block is composed of an array of 4 × 2 detection units. Each detection unit consists of an array of 5 × 5 lutetium oxyorthosilicate cerium-doped (LSO:Ce) crystal elements of 3.2 × 3.2 × 20 mm size coupled with a SiPM detector with a dimension covering the entire scintillation area [17]. The camera was cross-calibrated with the two-dose calibrators used for the preparation of radioactive solutions in this study: a Unidose dispenser (Trasis, Ans, Belgium) equipped with a VIK-202 dose calibrator (Comercer, Joure, Netherlands) for 18F and a shielding hot cell equipped with a CRC-55tR dose calibrator (Capintec, Florham Park, New Jersey) for 68Ga.

### PET/CT acquisition and reconstruction

**Fig. 1:**
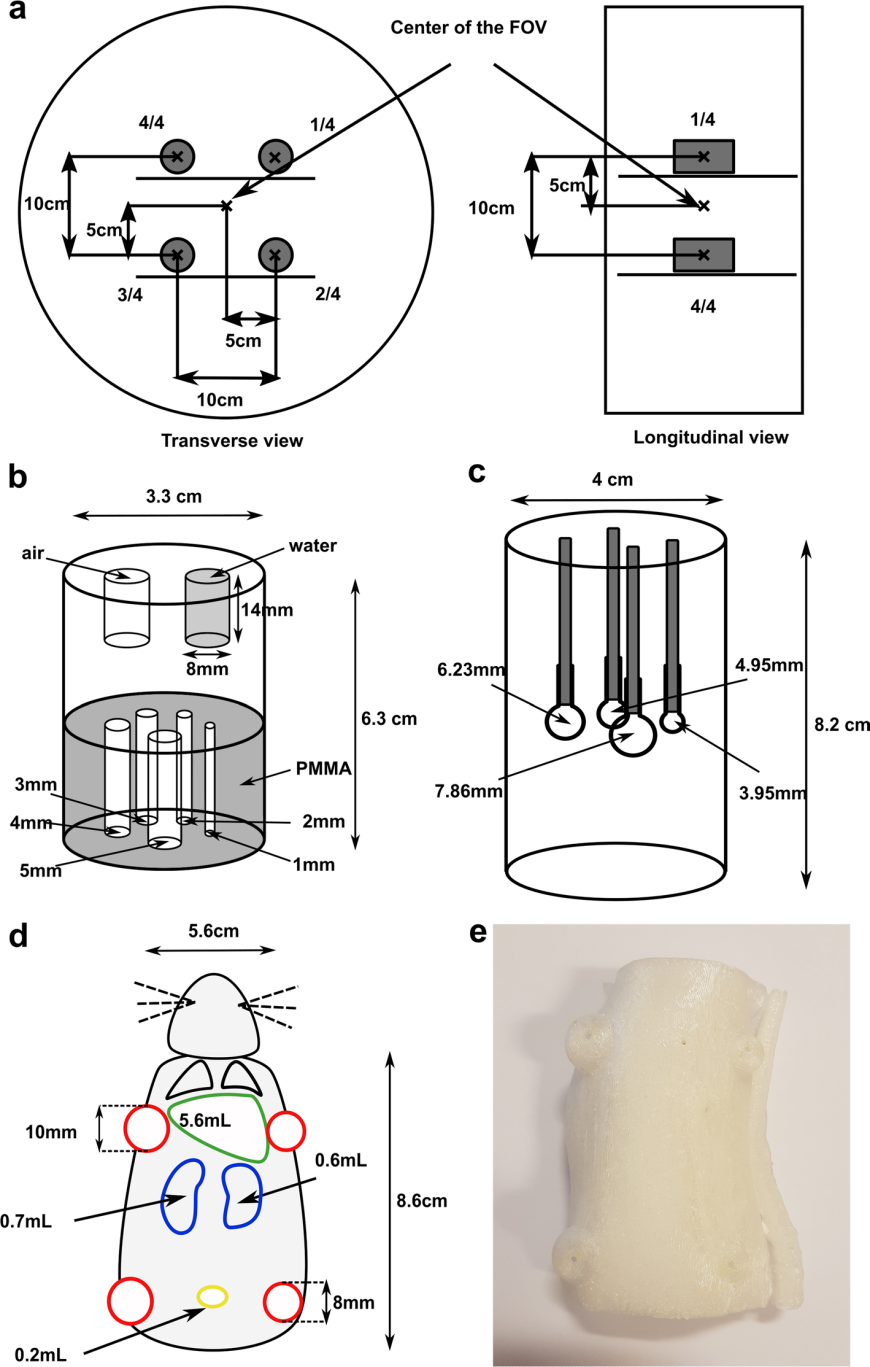
Diagram showing the different phantom locations for acquisitions mimicking the presence of 4 animals in the FOV seen in transverse and longitudinal planes (**a**) and drawings of the NEMA NU 4-2008 image quality phantom (**b**), Micro-hollow sphere phantom (**c**) and 3D-printed rat phantom (**d**) with the corresponding picture (**e**)

Twenty-minute emission scans in one bed position were performed with list mode data acquisition and TOF measurement. To assess the impact of imaging 4 rats simultaneously on image quality and quantification analysis using appropriate phantoms, the tests were repeated for two configurations, as follows: first the measurement phantoms were acquired in a centred position in the FOV without a scattering source (C-SC^−^) and then in the off-centre position at a radial and tangential offset of 5 cm, with 3 additional scattering sources to mimic the presence of 4 rats (OC-SC^+^), as illustrated in Fig. 1a. The scattering sources used in the experiments were fillable cylinders with a height of 90 mm and an internal diameter of 4 mm. All experiments were carried out for both 18F and 68Ga radionuclides.

CT acquisitions were performed for attenuation correction purposes with the following parameters: tube voltage of 80 kV, tube current of 23 mA, table moving speed set to a pitch of 1, total collimation aperture of 19.2 mm, slice thickness of 1.5 mm and a matrix size of 512 × 512.

Images were reconstructed for attenuation and scatter diffusion correction with a zoom factor of 2 and a matrix size of 880 × 880. The resulting pixel size of the reconstructed images was 0.4125 × 0.4125 mm. Depending on the test performed, images were reconstructed using 3 different algorithms: filtered backprojection (FBP), 3D-ordered subset expectation maximization algorithm (3D-OSEM) and vendor-proprietary iterative algorithm modelling PSF (TrueX) using 5 iterations (i) and varying the number of subsets (s) from 3 to 30.

### Intrinsic spatial resolution

The spatial resolution of the system was assessed using capillaries with a 1 mm internal diameter filled with an 18F solution of 0.5 MBq/mL. Two emission scans of 10 min duration were performed with capillaries placed first in the centre of the FOV and then in position 1/4 (Fig. 1a), corresponding to the off-centre position. Images were reconstructed with the FBP, 3D-OSEM and PSF algorithms as described previously in the PET/CT section. Profiles passing through the maximum pixel value of each source were drawn in the radial, tangential and axial directions using AMIDE software [18]. The full width at half maximum (FWHM) and the full width at tenth maximum (FWTM) in mm were determined in each direction using linear interpolation between adjacent pixels at half and one-tenth of profile’s maximum value, respectively. The maximum value was determining using a parabolic adjustment of the peak, as recommended by the NEMA NU 4-2008 standard.

### Image quality

Image quality was assessed using a NEMA NU 4-2008 PET image quality phantom (Fig. 1b). This phantom consists of a fillable chamber with a height of 63 mm and an internal diameter of 33.5 mm and is composed of three different sections. The first part, containing two cylindrical inserts of 14 mm height and 8 mm inner diameter filled with air and cold water, respectively, was used to evaluate the spill-over ratio (SOR). A second part, free of structures, was used to evaluate the reconstructed image uniformity. A third part, containing a solid PMMA insert drilled with 5 holes of 5, 4, 3, 2 and 1 mm inner diameter, was used to evaluate recovery coefficients (RC).

The phantom was filled with either 18F or 68Ga solution of approximately 3.7 MBq of activity at the start of acquisition. Two emission scans of 20 min duration were performed for C-SC^−^ and OC-SC^+^ acquisitions. The activity prepared for the 3 scattering sources was approximately 4 MBq. The time duration for the second acquisition was adapted to consider the radiation decay.

To assess uniformity, a cylindrical VOI 22 mm in diameter and 10 mm in length was drawn in the uniform part of the phantom. The average, minimum, maximum and standard deviation (SD) were measured.

Images were reformatted to obtain 10-mm-thick slices centred on the hot rods. Circular ROIs encompassing each rod were drawn, with dimensions twice the physical size of the rods. The maximum pixel value of each ROI was measured, and the RC was calculated as follows:

$${RC}_{rod}=\frac{max\,pixel\,\,value\,\,of\,\,the\,\,rod}{mean\,\,pixel\,\,value\,\,in\,\,the\,\,uniform\,\,part}$$

Line profiles (10 mm) were drawn for each rod in the axial direction, passing through the coordinate of the maximum pixel value measured previously. The mean (Mean_lineprofile_) and SD (SD_lineprofile_) of the pixel values measured along the profile were measured, and the SD of the RC were calculated as follows:

$${SD}_{RC}=RCx\sqrt{({\frac{{SD}_{lineprofile}}{{Mean}_{lineprofile}})}^{2}+({\frac{{SD}_{background}}{{Mean}_{background}})}^{2}}$$

With Mean_background_ and SD_backgroud_, the mean and SD values were measured in the uniform part.

VOIs of 4 mm diameter and 7.5 mm length were drawn on the air and water insert to calculate the spill-over ratio as follows:

$${SOR}_{water}=\frac{{Mean}_{water}}{{Mean}_{background}}$$

$${SOR}_{air}=\frac{{Mean}_{air}}{{Mean}_{background}}$$

All measurements were performed in accordance with the NEMA NU 4 standards.

### Quantification accuracy

To evaluate quantification accuracy, RCs were computed using a hot spot phantom equivalent to a small animal in size, filled with an activity concentration ratio between spheres and background. The phantom used for this test was a micro-hollow sphere (MHS) phantom that consists of a fillable cylinder with an internal diameter of 4 cm and a height of 8.2 cm, containing 4 hollow spheres with inner diameters of 7.86, 6.23, 4.95 and 3.95 mm (Fig. 1c). Spheres and background were filled with either 18F or 68Ga solutions. For the F18-filled phantom, two different sphere-to-background ratios were tested, filling the background with an activity of 4 MBq at the start of acquisition and the spheres with an activity concentration of 0.3 MBq/cc to obtain a contrast ratio of 1/8 or 0.14 MBq/cc for a ratio of 1/4. For the 68Ga experiment, only the 1/8 contrast ratio was tested. For the image quality phantom experiment, C-SC^−^ and OC-SC^+^ acquisitions were performed to test the impact of imaging 4 rats simultaneously. Images were reconstructed using 3D-OSEM and PSF algorithms by varying the total number of iterations to determine the optimal PET reconstruction settings for quantification purposes.

To determine the RC values, the method proposed in the EANM procedure guidelines for FDG tumour PET imaging was used [19]. A cylindrical region of interest (ROI) was drawn in the uniform part of the phantom to determine the mean activity in the background. Four spherical volumes of interest (VOIs) encompassing the hot spheres were drawn to determine the maximum pixel value of each sphere. 3D isocontours at 50% of the maximum value adapted for background activity measured in the uniform part were delineated to determine the mean activity in the spheres (A50).

RC max and RC A50 were calculated for each sphere as follows:

$$RC max=\frac{Maximum\, pixel \,value\, (Bq.{mL}^{-1})}{Theoretical\, activity\, in \,sphere\, \left({Bq.mL}^{-1}\right)}$$

$$RC A50=\frac{Mean\, activity\, in\, the\, isocontour\, at 50\%\, of \,the \,maximum\, pixel\, value\, \left({Bq.mL}^{-1}\right)}{Theoretical\, \,activity \,in \,sphere \,\left({Bq.mL}^{-1}\right)}$$

### Validation of quantification using a 3D-printed anatomical rat phantom

To test quantification in preclinical conditions, four 3D-printed rat phantoms represented in Fig. 1d, e were used. These phantoms were modelled by manually contouring the main organs visible on CT images of a real rat previously imaged on a preclinical CT device by our team as described in a previous study [20]. The resulting rat phantoms consisted of two air cavities simulating the lungs and four fillable cavities of 5.6, 0.2, 0.6 and 0.7 mL simulating the liver, bladder (only for two of the four rats), right and left kidneys, respectively. Four spherical cavities with two different sizes were added to the upper left, upper right, bottom left and bottom right of the rat phantom to mimic the presence of pertinent sized subcutaneous tumours. The two tumours located on the left side of the rat had an internal diameter of 10 mm corresponding to a volume of 0.53 mL, and the two tumours located on the right side had a diameter of 8 mm corresponding to a volume of 0.27 mL. The phantoms were printed with a Tevo Tornado 3D printer (Tevo 3D Electronic Technology Co. Ltd., China) using polyethylene terephthalate glycol plastic as the printed material.

The liver, kidneys and bladder were filled with 18F solutions of 0.3, 1.2 and 2.4 MBq/mL at the start of acquisition. Tumours of the rats were filled with different activity concentrations to obtain different contrast ratios between tumours and organs. The four tumours were filled with 0.3, 0.6, 1.2, and 2.4 MBq/mL of 18F. The tumour locations of the different radioactive concentrations were alternated for each rat. Twenty-minute acquisitions were repeated five times every 30 min to simulate a tumour activity reduction. The resulting total activity present in the four rats varied approximately from 5.7 MBq to 2.85 MBq from the first to the last acquisition. The same experiment was repeated by filling the rats with 68Ga in place of 18F. A total of 80 tumours with variations in size, location and activity concentration were thus quantified in this study for both radionuclides. Images were reconstructed using the PSF algorithm with 20 i and 5 s. For 18F acquisitions, a 2-mm FWHM Gaussian filter was applied in addition to complying with the best reconstruction settings determined with the MHS phantom. For each tumour visible on the different acquisitions performed, the quantification parameters Amax and A50 were determined as described in the recovery coefficient section and compared to the theoretical activity concentration (Acalc) calculated at the acquisition start.

## Results

### Intrinsic spatial resolution

Table 1 shows the radial, tangential and axial FWHM and FWTM calculated for FBP, 3D-OSEM and PSF reconstructions with the source placed at the two tested positions in the FOV. For 3D-OSEM and PSF reconstructions, the results are only presented for 20 i and 5 s, for which the convergence of the algorithms has been reached. The results obtained for all other reconstruction settings are presented in Additional file 1: Table S1. The FWHM measured at the centre of the FOV using the PSF reconstruction algorithm was 1.80 mm in the axial direction. However, for 3D-OSEM and FBP reconstructions it increased to 3.13 mm and 3.20 mm, respectively. When the source was positioned in off-centre position in the FOV, the FWHM measured in the radial direction for PSF reconstruction was 1.78 mm. Conversely, FWHM increased to 3.59 mm and 4.20 mm for 3D-OSEM and FBP reconstructions, respectively.

**Table 1:** Spatial resolution obtained in the centre of the FOV and in the off-centre position for different reconstruction settings. Radial, tangential and axial resolutions are expressed as FWHM (FWTM) in mm

	Centre			Off-centre		
	Radial	Tangential	Axial	Radial	Tangential	Axial
FBP	3.86 (7.03)	3.90 (7.08)	3.91 (7.13)	4.20 (7.69)	4.23 (7.77)	4.22 (7.78)
3D-OSEM	3.13 (5.68)	3.17 (5.78)	3.17 (5.77)	3.59 (6.56)	3.62 (6.60)	3.67 (6.64)
PSF	1.80 (3.35)	1.79 (3.28)	1.85 (3.48)	1.78 (3.22)	1.79 (3.27)	1.81 (3.50)

### Image quality

**Fig. 2:**
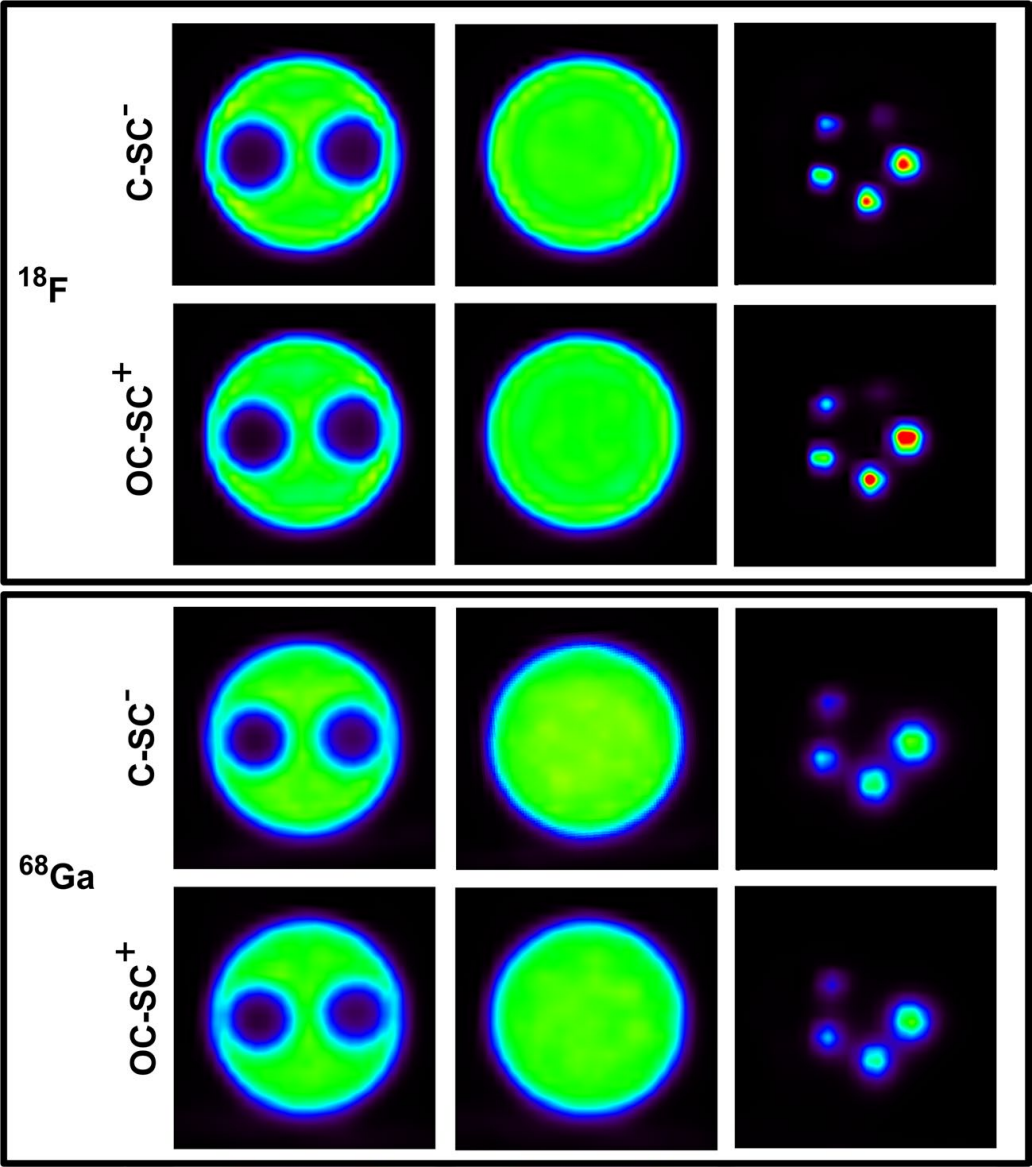
Image samples of 18F and 68Ga-filled NEMA NU 4-2008 phantoms performed for C-SC^−^ and OC-SC^+^ acquisitions and reconstructed using the PSF algorithm with 20 iterations and 5 subsets. Axial slices are centred in the three different parts of the phantom: air and water inserts (left), uniform region (middle) and capillaries (right). The image window was normalized on the background of the phantom

**Fig. 3:**
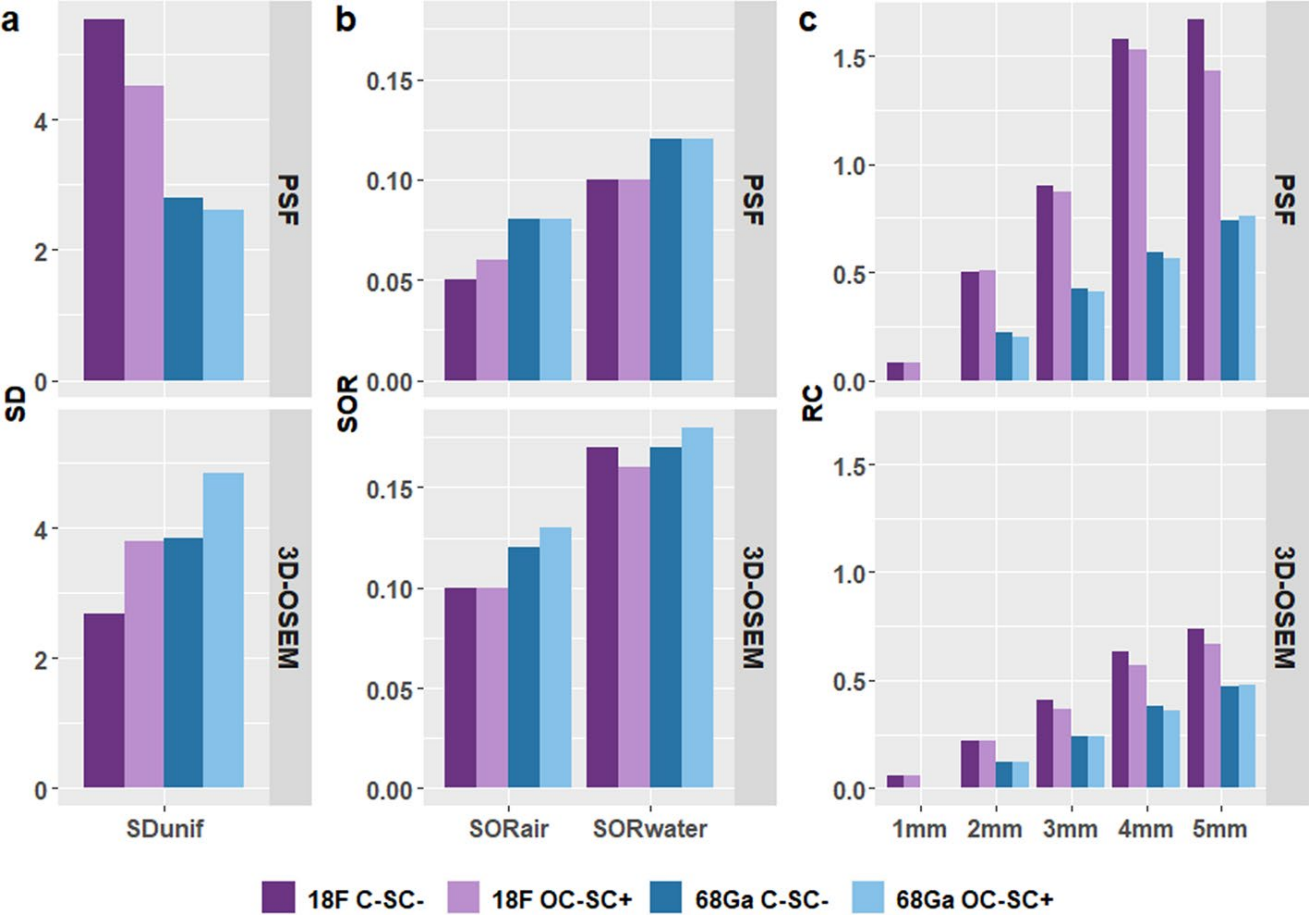
Uniformity (**a**), SOR (**b**) and RC values (**c**) calculated for 18F and 68Ga-filled NEMA U4-2008 phantoms performed for C-SC^−^ and OC-SC^+^ acquisitions and reconstructed with 3D-OSEM and PSF algorithms

Sample images of the NEMA NU 4-2008 phantom are presented in Fig. 2 for C-SC^−^ and OC-SC^+^ acquisitions and both radionuclides tested in this study. Additional images of the phantom comparing different reconstruction settings are presented in Additional file 2: Figure S1. Figure 3 shows SORair, SORwater, uniformity and RC values calculated on phantom images for the corresponding acquisitions. Only results obtained for OSEM and PSF reconstructions with 20 i and 5 s are presented. The results obtained for all reconstructions tested are presented in Additional file 1: Tables S2 to S5.

Regarding C-SC^−^ acquisitions, the SOR values computed for the 18F-filled phantom were 0.05 in air and 0.10 in water for the PSF reconstruction, and 0.10 in air and 0.16 in water for the 3D-OSEM reconstruction. These SOR values increased for the 68Ga-filled phantom to 0.08 in air and 0.12 in water for PSF and 0.12 and 0.17 in water for 3D-OSEM.

The uniformity values (%SD) computed for the 18F-filled phantom were 5.55% for PSF and 2.69% for the 3D-OSEM reconstruction. For the 68Ga-filled phantom, the uniformity values were 2.81% for PSF reconstruction and 3.84% for 3D-OSEM reconstruction.

The RC values were computed for the 18F-filled phantom, ranging from 0.08 to 1.67 for PSF reconstruction, depending on the size of the spheres. In contrast, for 3D-OSEM reconstruction, the RC values decreased from 0.06 to 0.074. In the case of the 68Ga-filled phantom, the RC values were notably lower, with a maximum RC value of 0.74 for PSF reconstruction and 0.47 for the 3D-OSEM algorithm. It is important to note that the RC values were not calculated for the 1-mm rod in the 68Ga acquisitions, as it was not visible in the image.

Regarding the OC-SC^+^ acquisitions, we found that SOR values calculated for both 18F and 68Ga acquisitions were not different than for C-SC^+^ acquisitions. However, we noted a reduction of up to 10% in the uniformity and RC values compared to the C-SC^−^ acquisitions.

### Quantification accuracy

**Fig. 4:**
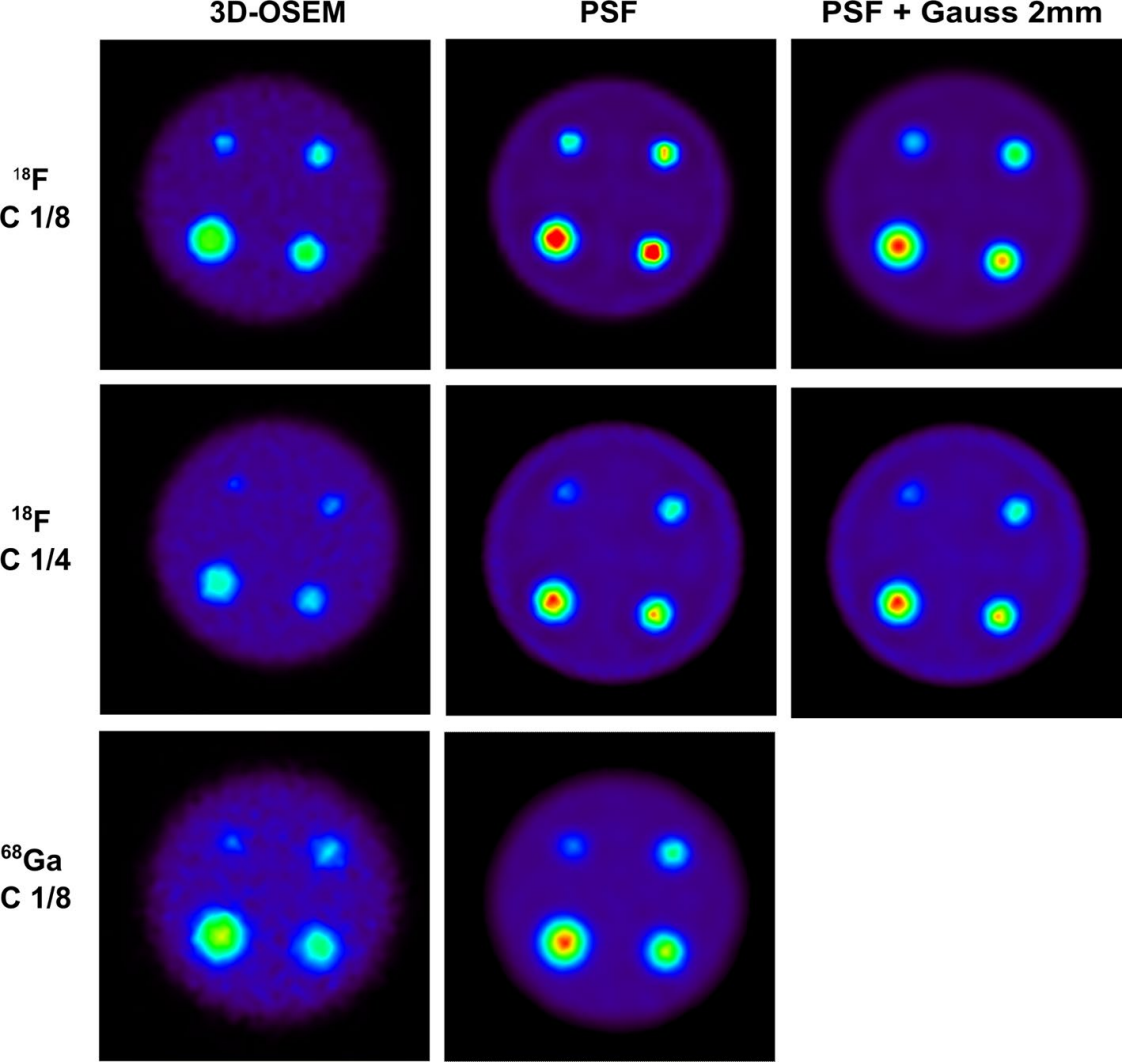
Axial slices of the MHS phantom positioned in the centre of the FOV for 18F with contrast 1/8 (top), F18 with contrast 1/4 (middle row) and 68Ga with contrast 1/8 (bottom row). Three different reconstruction settings are presented: 3D-OSEM (left column), PSF (middle column) and PSF with a 2-mm FWHM Gaussian filter (right column). The image window was normalized on the background of the phantom

**Fig. 5:**
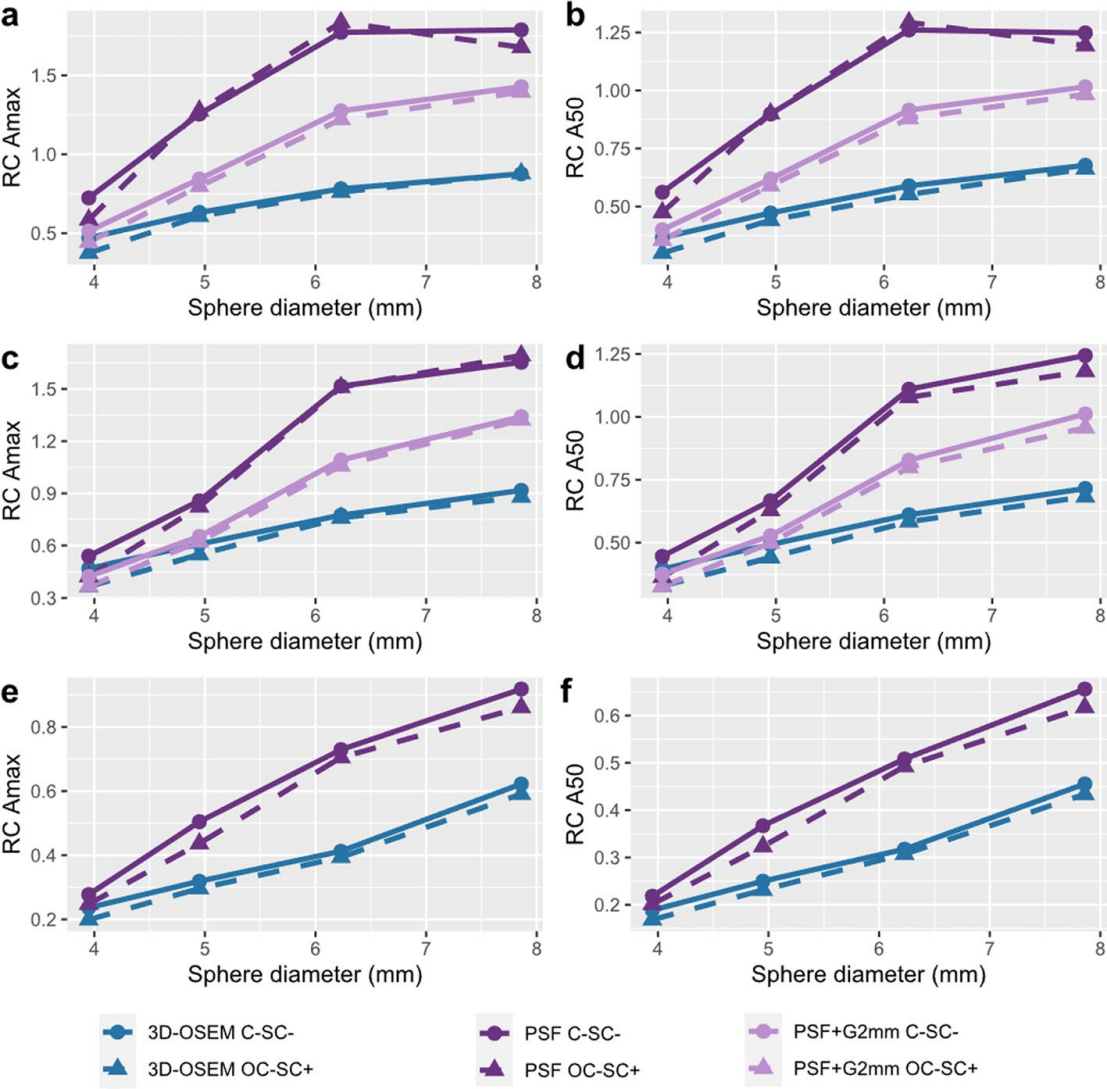
Recovery coefficients calculated using the MHS phantom as a function of sphere size for Amax (left column) and A50 (right column). The results are presented for the 18F-filled phantom with 1/8 (**a**, **b**) and 1/4 (**c**, **d**) sphere-to-background contrast ratios and for the 68Ge-filled phantom with a 1/8 contrast ratio (**e**, **f**). For each graph, RC values were computed for C-SC^−^ and OC-SC^+^ acquisitions and for the different reconstruction settings

Sample images obtained with the MHS phantom are presented in Fig. 4. The RC values calculated for C-SC^−^ and OC-SC^+^ acquisitions as a function of sphere sizes are presented in Fig. 5. The results are presented for 3D-OSEM and PSF reconstructions with both 20 i and 5 s. For the PSF reconstruction, a second image data set was reconstructed using a 2-mm FWHM Gaussian post-reconstruction filter. All recovery coefficients calculated for the different reconstruction algorithms and iteration settings are presented in Additional file 1: Tables S6 to S8. For all acquisitions performed, we observed a decrease in RC max and RC A50 with sphere size. Comparing RC max and RC A50 values calculated for C-SC^−^ and OC-SC^+^ acquisitions, the differences did not exceed 5%, regardless of the reconstruction method used and for both 18F and 68Ga acquisitions.

For the 18F-filled MHS phantom reconstructed with PSF modelling and without a post-reconstruction filter, we observed an overestimation of the recovery coefficients for the two quantification parameters, with values ranging as a function of sphere size from 0.64 to 1.65 for RC max and from 0.45 to 1.24 for RC A50. This quantitative bias was reduced by applying a 2-mm FWHM Gaussian filter to the PSF reconstruction with 20i and 5 s, which allowed us to obtain values ranging from 0.42 to 1.34 for RC max and from 0.38 to 1.01 for RC A50 as a function of sphere diameter.

For the 68Ga-filled MHS phantom, the RC max and RC A50 values were lower than those obtained with 18F. Values obtained for the PSF reconstruction with 20 i and 5 s ranged as a function of sphere size from 0.30 to 0.80 for RC max and from 0.24 to 0.64 for RC A50.

### Validation of quantification using a 3D-printed anatomical rat phantom

**Fig. 6:**
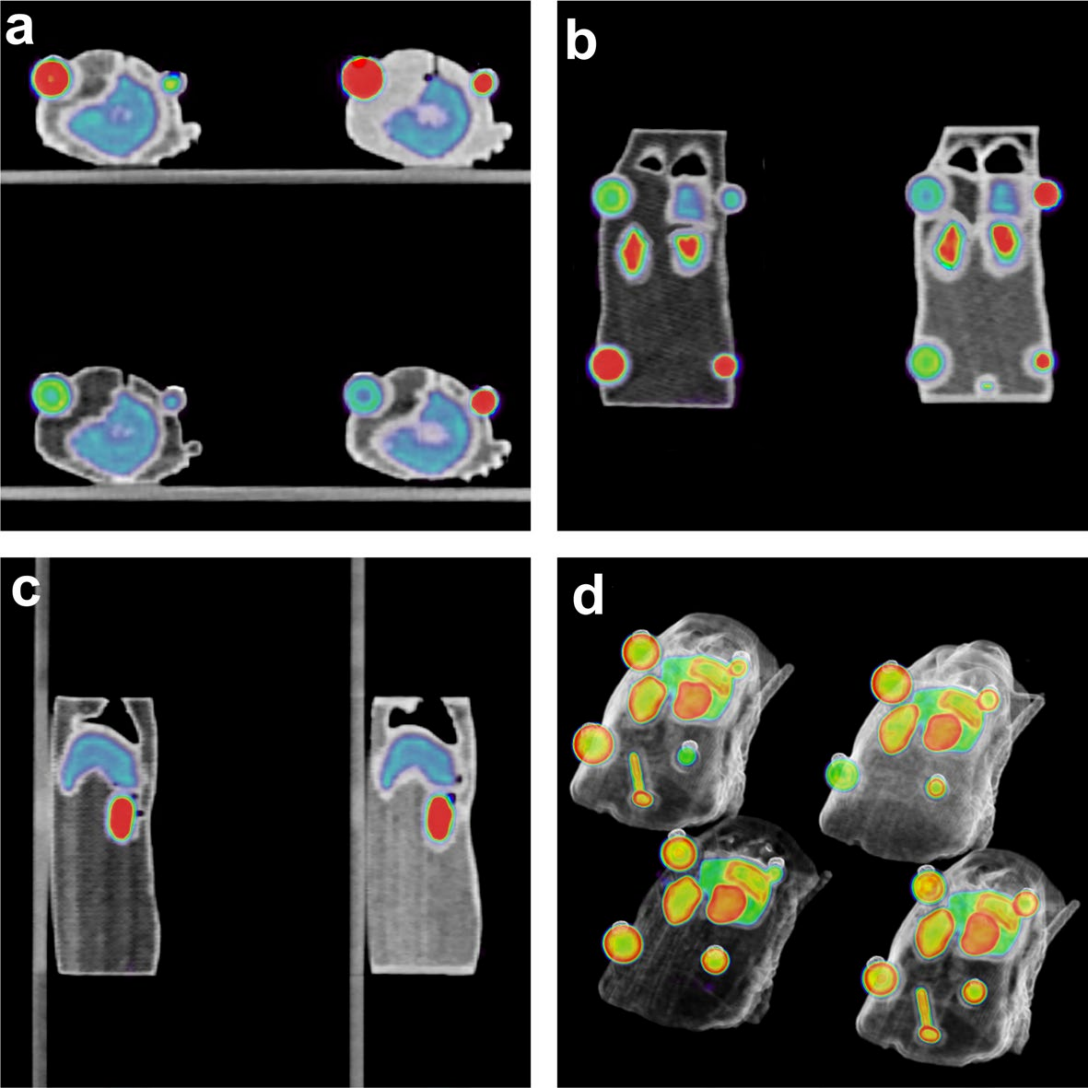
PET-CT images of the four 3D-printed rat phantoms filled with F18. Representative images are displayed in transverse (**a**), coronal (**b**) and sagittal (**c**) views as well as in 3D using a volume rendering technique (**d**)

**Fig. 7:**
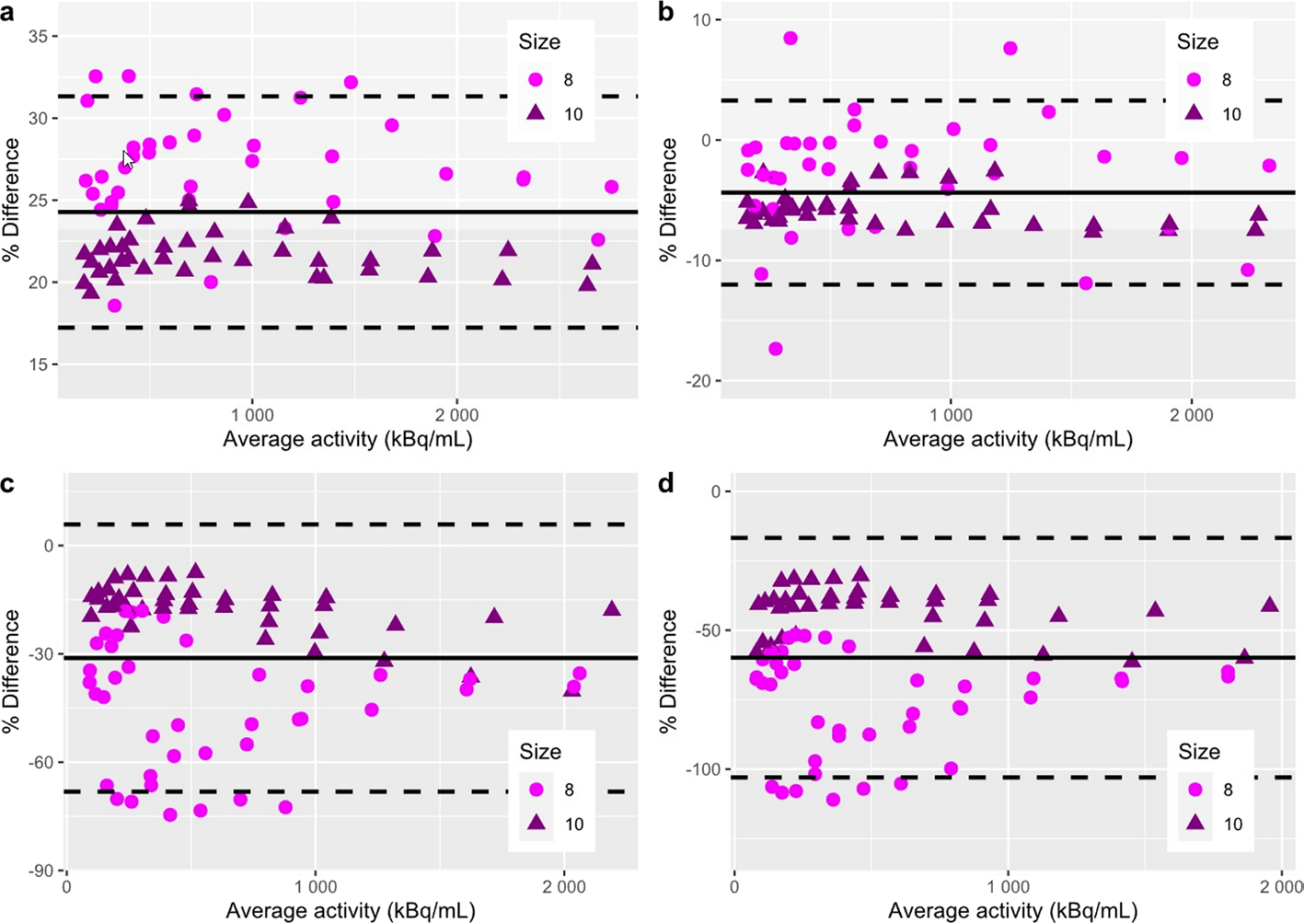
Bland‒Altman plots of per cent difference obtained for 18F (**a**, **b**) and 68Ga (**c**, **d**) acquisitions. The results are presented for Amax (left) and A50 (right) as a function of sphere size

Figure 6 shows representative fused PET/CTimages obtained for the acquisition of four rats simultaneously. The CT images of the four 3D-printed rat phantom, filled with water, are presented in Additional file 2: Fig. 2 to show the achieved image quality on anatomical data under low contrast conditions. For 18F acquisitions, Pearson correlation coefficients between the measured and calculated activity for each tumour were equal to 0.9976 and 0.9982, respectively, for Amax and A50, showing good agreement. These values were equal to 0.9886 and 0.9794 for the 68Ga acquisition. Bland‒Altman plots presented in Fig. 7 show that, for both 18F and 68Ga acquisitions, the activity measurements on tumours were globally included in the interval of confidence, which demonstrated good measurement accuracy. However, we observed that the dispersion of the measured values was more important for the 8-mm tumours, particularly when the activity decreased. Finally, it can be noted that few measurement points were outside the confidence interval. This was due to a slight leakage observed on the CT images for one of the smallest tumours of a rat, which led to a measurement bias related to the small volume remaining in the cavity.

## Discussion

New digital technologies using SiPM-based detectors have significantly improved the intrinsic performance of clinical PET devices. In particular, this technology has improved the measured signal-to-noise ratio, allowing refinement of the image matrix size (880 × 880 against 440 × 440 for analogical PET devices), which is associated with a higher spatial resolution [10, 21]. This is a critical point for imaging small animals, where the limiting factor is the capacity of the system to detect millimetre-sized structures within a small volume of distribution.

The aim of this study was to assess the feasibility of using such a PET system for tumour imaging in small-animal with for preclinical research purposes. Spatial resolution measurements carried out using line sources demonstrated the system's ability to image such structures. Especially, the use of a PSF modelling algorithm significantly improved these measurements in comparison with FBP or 3D-OSEM reconstruction method, resulting in a computed FWHM of 1.80 mm at the centre of the FOV. The results also indicated that the PSF modelling led to a more uniform resolution throughout the field of view in contrary to the other reconstructions method, when comparing FWHM computed in centre and off-centre position. Therefore, the use of this reconstruction method appears essential for preclinical applications in which spatial resolution is a critical factor.

The assessment of image quality also indicated the system’s ability to perform small-animal imaging, as evidenced by visual assessment of the three different parts of the NEMA NU 4 phantoms. This was especially noticeable when using PSF reconstruction, which resulted in improved visibility of rods and enhanced contrast in air and water cavities. Specifically, the RC values calculated for the rod of different diameters were higher compared to the 3D-OSEM reconstruction, meaning better spatial resolution. SOR calculated in air and water were much smaller than those obtained with 3D-OSEM reconstruction, highlighting the PSF reconstruction method’s superior correction of the spill-over effect. These findings support the necessity of employing such an algorithm for image reconstruction.

To assess whether the performance achieved with this system is sufficient, it may be interesting to compare the results with those obtained from dedicated SA-PET cameras. Table 2 compares the results obtained in this study with published performance tests assessed according to the NEMA NU 4-2008 standards on SA-PET devices available in the early 2010s [22–26] as well as for state-of-the-art cameras [27–29]. We found that the intrinsic performances reached by a recent PET clinical device in terms of resolution and image quality were very close to that of earlier generations of SA-PET cameras. The intrinsic resolution obtained with the SiPM-based system associated with PSF image reconstruction was thus in the same range, with a measured radial FWHM value of 1.80 mm at the centre of the FOV. However, more recent SA-PET devices demonstrates significantly better results, with measured FWHM of less than 1 mm. As the reconstructed NEMA NU 4–2018 phantom with the PSF modelling algorithm showed RC values much higher than 1, an additional image data set was reconstructed with a 2-mm FWHM Gaussian post-filter for regularization purpose. Corresponding image quality parameters were computed, and the results are added in Table 2. The resulting SOR values calculated for water and air were equal between filtered and unfiltered PSF reconstructions, while the uniformity value improved by approximately 40%. Compared to the dedicated SA-PET cameras available in the 2010s, the RC values calculated for the clinical SiPM-based PET camera are of the same order. We could only note a significantly lower RC value for the 1-mm rod. On the other hand, the contrast level achieved by state-of-the-art SA-PET cameras demonstrates a general enhancement for all spheres.

**Table 2:** Comparison of spatial resolution and image quality parameters obtained between dedicated SA-PET devices and Vision clinical SiPM-based PET system

	MicroPET focus 120 [22]	MosaicHP [23]	Inveon [24]	Flex triumph [25]	Clear PET[26]	rPET [26]	NanoScan [27]	Iris [28]	PET Insert Si 198 [29]	Biograph Vision 450	
										PSF	PSF + G2 mm
Radial FWHM centre FOV (mm)	1.92	2.32	1.81	~ 1.8	1.9	1.4	1.5	1.05	0.75	1.80	-
Radial FWTM centre FOV (mm)	3.66	5.3	3.57	~ 3.6	3.8	2.5	3.29	2.35	1.38	3.35	-
SORwater	0.018	0.063	0.046	0.09	0.27	0.15	0.097	0.11	0.22	0.10	0.10
SORair	0.003	0.027	0.047	0.1	0.37	0.24	0.086	0.11	0.12	0.05	0.05
Uniformity (%SD)	6	5.1	7.447	6.01	-	-	6.53	7.0	6.5	5.54	3.47
RC 5 mm	0.93	0.84	1.059	0.89	0.89	0.81	0.92	0.89	0.94	1.67	1.1
RC 4 mm	0.86	0.7	0.911	0.68	0.73	0.76	0.8	0.82	0.95	1.58	0.91
RC 3 mm	0.75	0.56	0.816	0.56	0.42	0.66	0.65	0.73	0.91	0.9	0.56
RC 2 mm	0.48	0.36	0.604	0.43	0.21	0.46	0.49	0.58	0.64	0.5	0.28
RC 1 mm	0.15	0.16	0.367	0.15	0.14	0.11	0.24	0.14	0.14	0.08	0.06

The sensitivity of a PET system is another important parameter to consider when assessing its capacity to detect low levels of activity. Although the sensitivity defined in the NEMA NU 4 standards for preclinical PET imaging systems was not specifically assessed in this study, we can compare the sensitivity values reported in the literature for the specific camera model used in our study. Carlier et al. [10] measured a total sensitivity of 9.5 counts/MBq for an energy window of 435 to 585 keV, which corresponds to a total absolute sensitivity of 0.94%. It is important to note that this sensitivity was assessed following the NEMA NU2 standards and utilized an 18F linear source instead of the 22Na point source recommended in the NEMA NU 4 standards. When comparing the total absolute sensitivity values reported in the literature for the SA-PET cameras listed in Table 2, which vary from 1 to 10%, the sensitivity measured on the clinical PET system in our study falls within the lower end of this range. This can be attributed to a detection geometry that is less optimized for small-animal imaging. However, it is worth mentioning that all the phantoms used in our study were filled with the activity recommended in the NEMA NU 4 standards, which corresponds to the activity levels commonly used in preclinical practice. The favourable results obtained with this level of activity suggest that the overall sensitivity of the system is sufficient for our intended preclinical applications.

We have seen that the intrinsic performance of the camera greatly depends on the reconstruction settings used. As a consequence, performance measurements such as FWHM, SOR in air and water, uniformity or RC values measured with the NEMA NU 4-2008 and MHS phantoms can be impacted by PSF modelling or the total number of iterations used for image reconstruction. As shown by the results, spatial resolution using PSF reconstruction increased with the total number of iterations used until it reached a plateau for a total number of 100 iterations, with a corresponding radial FWHM of 1.95 mm measured at the centre of the FOV. At the same time, we observed that the measured activity on the hot spheres of the MHS phantom filled with 18F was overestimated when using these reconstruction settings. Indeed, we computed an RC max and an RC A50 of 1.79 and 1.25, respectively, for the largest spheres of the MHS phantom centred in the FOV with a contrast ratio of 1/8 between the spheres and background. Thus, while the use of PSF modelling can improve spatial resolution, it can lead to a quantitative bias with an overestimation of activity measured in tumours, especially for noise-sensitive parameters such as RC max [30]. As a result, RC values calculated on the NEMA NU 4-2008 were largely higher than one for the largest sphere when using PSF reconstruction. If PSF modelling improves visual image analysis with contrast enhancement and better lesion detectability, its use without an additional post-reconstruction filter is not necessarily suitable for PET quantification. On the other hand, we have shown that the results obtained with 3D-OSEM reconstructions were not optimal for accurate quantification. Indeed, the RC calculated for the largest sphere of the 18F-filled MHS phantom did not exceed 0.88 for Amax and 0.68 for A50 with a contrast ratio of 1/8 between the spheres and background. To solve this issue, we have proposed reconstructing two image data sets as proposed by the EARL recommendations for 18-FDG in human patients [31, 32]: one reconstruction using PSF modelling can be reconstructed for visualization purposes only, and a second image data set can be reconstructed with a suitable Gaussian filter for quantification purposes only. The choice of the post-reconstruction filter to be used can be determined using the MHS phantom so that the RC value calculated for A50 on the largest sphere approaches the theoretical value of 1. This methodology can also be used to harmonize quantitative results obtained on different systems. Such a solution could be implemented on all equipment used in preclinical research, including the latest generation devices dedicated to small-animal imaging, to better consider equipment heterogeneity and obtain reproducible quantitative results regardless of the device used.

The development of molecularly targeted therapies can potentially lead to an increase in the number of groups to be imaged: Aide et al. [33] have reported that as many as 45 mice would be needed for an experiment in which five groups of mice are to be imaged and a 30% decrease in tracer uptake is expected. As the number of animals that must be imaged during a preclinical PET experiment increases, there is a need to improve image throughput, which can be accomplished by imaging several animals simultaneously [33]. Some authors have already tested the feasibility of imaging four mice simultaneously on a dedicated SA-PET device with a large bore [34]. For this experiment, a custom bed holder was developed to accommodate 4 animals within the PET system’s the bore. However, due to the size limitations of the bed holed, it was not possible to image animals larger than a mouse. In a more recent study by Efthimiou et al. [35], the impact of scanning multiple animal on image quality was investigated. The authors found that imaging more the two animals simultaneously resulted in a loss of image quality. The advantage of clinical PET systems, compared to SA-PET devices, is their large field of view, which makes the simultaneous acquisition of several animals easier. In a previous study, we were able to show the possibility of imaging four mice at the same time on an analogue clinical PET scanner [9]. It is also important to assess the impact of having multiple animals in the field of view at the same time on quantification. The results have shown the low impact on spatial resolution, image quality and quantification assessment when imaging phantom in off-centred position with three other scattering sources in the FOV. This suggests the possibility of imaging four animals simultaneously without compromising the quality of visual and quantitative image analysis.

Conversely, we observed an impact on image quality and quantification when imaging phantoms with 68Ga instead of 18F. We have thus observed a greater partial volume effect on phantom images, leading to higher values of SOR in air and water and a decrease in spatial resolution, leading to a lack of visibility of the 1-mm rod on the NEMA NU 4-2008 phantom. As reported by Disselhorst et al. [24], a larger SOR in water could be attributed to the longer positron range for 68Ga compared to 18F, which leads to positrons being emitted in the body part of the phantom but annihilated in the cold water compartment. This has an equally important impact on PET quantification, as revealed by RC values obtained with the MHS phantom, which were inferior to those obtained with 18F regardless of the size of the spheres. Nevertheless, the performances can be considered satisfactory with regard to the results obtained with the MHS phantom as well as with the 3D-printed phantom. Indeed, all the hot spheres of the MHS phantom and all the tumour sizes of the 3D-printed phantom were perfectly visible on images with a sufficient contrast ratio.

We have finally demonstrated in this study that it was possible to achieve quantitative preclinical PET imaging, even under disturbing conditions such as the presence of several animals in the FOV or the use of long positron range radionuclides. Imaging of small structures makes this quantitative data analysis difficult, with the presence of a partial volume effect for tumours of size approaching the intrinsic spatial resolution of the system. The gain in spatial resolution and contrast enhancement provided by the digital camera as well as the use of PSF modelling for image reconstruction have thus contributed to decreasing the impact of these quantitative measurement biases and reaching a sufficient level of accuracy for preclinical research in oncology. The measurements conducted using the 3D-printed rat phantom demonstrated that it was possible to achieve accurate and reproducible quantification results. This was achieved by varying parameters such as the size and activity of the tumours, the tumour-to-organ ratios, and the overall activity level within the field of view.

In this study, we did not assess the performance of the CT scanner integrated in the Vision 450 system. Indeed, CT acquisitions were only used for attenuation correction of PET images. The images presented in Additional file 2: Fig. 2 show a good spatial resolution when imaging the four rat phantoms simultaneously, allowing clear differentiation of organ. Although the achieved spatial resolution adequately meets attenuation correction requirements, it might be comparatively lower than the resolution achieved by CT scanners embedded into the most recent hybrid SA-PET systems. This disparity could possibly impose limitations based on the performance level expected by users.

This study was limited only to the technical feasibility of using a clinical PET camera to perform small-animal imaging. However, it is important to bear in mind the increasing difficulty in performing preclinical experiments on clinical PET-CT cameras, primarily driven by local regulations that prioritize patient care and by uphold ethical considerations and internal institutional policies. While using clinical PET-CT scanners for preclinical research has its limitations and challenges, it can still be a valuable tool in certain circumstances. Researchers must carefully consider the specific objectives of their studies, the availability of alternative preclinical imaging devices, and the ethical implications of utilizing human scanners. Collaborations between preclinical and clinical researchers can further optimize the utilization of human PET-CT scanners for preclinical investigations.

## Conclusion

Performances achieved with a clinical SiPM-based PET camera allowed the imaging of small animals injected with either 18F or 68Ga radionuclide for preclinical cancer research purposes. The image quality obtained was close to that obtained with dedicated SA-PET systems released in recent decades. Moreover, the extended field of view allowed us to image four rats simultaneously without loss of image quality. Finally, we showed accurate measurements of radioactive activity concentration carried out on subcentimetric lesions, which made tumour quantification in small animals using a clinical PET device possible.

### Supplementary Information

**Additional file 1.** Tables of results obtained for spatial resolution, image quality parameters and RC values calculated for various acquisition and reconstruction settings.

**Additional file 2.** SPECT images of the NEMA N4-2008 phantom and CT images of the 3D-printed rat phantoms.

## Data Availability

Data are available upon reasonable request.

## Abbreviations

3D-OSEM: 3D-ordered subset expectation maximization
C-SC^−^: Centred acquisition without scatter
FBP: Filtered backprojection
FWHM: Full width at half maximum
FWTM: Full width at tenth maximum
FOV: Field of view
HU: Hounsfield unit
LSO: Ce Lutetium oxyorthosilicate cerium doped
MHS: Micro-hollow sphere
NEMA: National Electrical Manufacturers Association
OC-SC^+^: Off-centred acquisition with scatter
PET: Positron emission tomography
PSF: Point spread function
RC: Recovery coefficient
ROI: Region of interest
SA-PET: Small-animal positron emission tomography
SiPM: Silicon photomultiplier
SOR: Spill-over ratio
SUV: Standardized uptake value
TOF: Time of flight
VOI: Volume of interest
