# Supplementary material for: PET imaging and quantification of small animals using a clinical SiPM-based camera
Source: EJNMMI Phys. 2023 Oct 7;10:61. doi: 10.1186/s40658-023-00583-2 (PMC10560240; doi:10.1186/s40658-023-00583-2)
Supplement: Supplementary file 1 — Additional file 1. Tables of results obtained for spatial resolution, image quality parameters and RC values calculated for various acquisition and reconstruction settings. [file 40658_2023_583_MOESM1_ESM.docx]

Table S1. FWHM and FWTM measured at the center of the FOV and with a 5cm radial and tangential offset. Radial, tangential and axial resolution measurements are expressed in mm and presented for the different reconstruction settings.

|  | Centre | | | | | | X=+5, y=+5 | | | | | |
| --- | --- | --- | --- | --- | --- | --- | --- | --- | --- | --- | --- | --- |
|  | Radial | | Tangential | | Axial | | Radial | | Tangential | | Axial | |
|  | FWHM | FWTM | FWHM | FWTM | FWHM | FWTM | FWHM | FWTM | FWHM | FWTM | FWHM | FWTM |
| FBP | 3,86 | 7,03 | 3,90 | 7,08 | 3,91 | 7,13 | 4,20 | 7,69 | 4,23 | 7,77 | 4,22 | 7,78 |
| 3D-OSEM i5s5 | 3,25 | 5,92 | 3,36 | 6,13 | 3,37 | 6,37 | 3,65 | 6,60 | 3,67 | 6,64 | 4,58 | 6,67 |
| 3D-OSEM i10s5 | 3,20 | 5,80 | 3,30 | 5,99 | 3,30 | 6,11 | 3,63 | 6,58 | 3,64 | 6,60 | 4,11 | 6,67 |
| 3D-OSEM i15s5 | 3,16 | 5,75 | 3,22 | 5,89 | 3,21 | 5,87 | 3,60 | 6,57 | 3,62 | 6,64 | 3,96 | 6,65 |
| 3D-OSEM i20s5 | 3,13 | 5,68 | 3,17 | 5,78 | 3,17 | 5,77 | 3,59 | 6,56 | 3,62 | 6,60 | 3,67 | 6,64 |
| 3D-OSEM i25s5 | 3,10 | 5,66 | 3,15 | 5,73 | 3,15 | 5,75 | 3,59 | 6,55 | 3,61 | 6,58 | 3,82 | 6,62 |
| 3D-OSEM i30s5 | 3,06 | 5,62 | 3,13 | 5,70 | 3,14 | 5,74 | 3,58 | 6,53 | 3,60 | 6,57 | 3,78 | 6,61 |
| PSF i5s5 | 2,29 | 3,92 | 2,19 | 3,70 | 2,37 | 3,86 | 2,16 | 3,89 | 2,24 | 4,03 | 2,24 | 4,16 |
| PSF i10s5 | 1,91 | 3,71 | 1,86 | 3,41 | 1,89 | 3,55 | 1,86 | 3,39 | 1,92 | 3,50 | 1,96 | 3,60 |
| PSF i15s5 | 1,85 | 3,53 | 1,83 | 3,32 | 1,86 | 3,50 | 1,79 | 3,24 | 1,82 | 3,32 | 1,84 | 3,36 |
| PSF i20s5 | 1,80 | 3,35 | 1,79 | 3,28 | 1,85 | 3,48 | 1,78 | 3,22 | 1,79 | 3,27 | 1,81 | 3,50 |
| PSF i25s5 | 1,79 | 3,34 | 1,78 | 3,25 | 1,84 | 3,45 | 1,77 | 3,20 | 1,77 | 3,22 | 1,79 | 3,27 |
| PSF i30s5 | 1,80 | 3,35 | 1,77 | 3,24 | 1,83 | 3,45 | 1,78 | 3,21 | 1,78 | 3,25 | 1,79 | 3,27 |

Table S2. Image quality parameters calculated for the 18F-filled NEMA NU 4-2008 phantom positioned in the center of the FOV (C-SC^-^). Results are presented for the different reconstruction settings tested.

| Recons | SOR_water_ | %SD water | SOR_air_ | % SD air | Uniformity %SD | RC 5mm | %SD 5mm | RC 4mm | %SD 4mm | RC 3mm | %SD 3mm | RC 2mm | %SD 2mm | RC 1mm | %SD 1mm |
| --- | --- | --- | --- | --- | --- | --- | --- | --- | --- | --- | --- | --- | --- | --- | --- |
| FBP | 0,18 | 11,49 | 0,11 | 20,45 | 1,74 | 0,64 | 1,85 | 0,49 | 2,25 | 0,29 | 2,82 | 0,16 | 4,19 | 0,04 | 13,17 |
| 3D-OSEM i3s5 | 0,24 | 5,98 | 0,19 | 7,44 | 1,39 | 0,77 | 2,28 | 0,64 | 2,06 | 0,39 | 2,65 | 0,20 | 3,07 | 0,03 | 8,91 |
| 3D-OSEM i5s5 | 0,20 | 6,56 | 0,15 | 8,73 | 1,75 | 0,76 | 2,77 | 0,63 | 2,42 | 0,41 | 2,69 | 0,22 | 3,11 | 0,04 | 7,31 |
| 3D-OSEM i10s5 | 0,17 | 7,29 | 0,13 | 9,76 | 2,21 | 0,74 | 3,53 | 0,62 | 2,92 | 0,41 | 3,15 | 0,22 | 3,56 | 0,06 | 6,74 |
| 3D-OSEM i15s5 | 0,17 | 7,91 | 0,10 | 12,85 | 2,49 | 0,74 | 3,94 | 0,63 | 3,17 | 0,41 | 3,59 | 0,22 | 3,85 | 0,06 | 6,84 |
| 3D-OSEM i20s5 | 0,17 | 8,51 | 0,10 | 14,29 | 2,69 | 0,74 | 4,21 | 0,63 | 3,33 | 0,41 | 3,90 | 0,22 | 4,06 | 0,06 | 6,94 |
| 3D-OSEM i25s5 | 0,17 | 9,08 | 0,10 | 15,47 | 2,85 | 0,74 | 4,38 | 0,63 | 3,46 | 0,41 | 4,15 | 0,22 | 4,22 | 0,07 | 7,06 |
| 3D-OSEM i30s5 | 0,17 | 9,60 | 0,10 | 16,46 | 2,98 | 0,74 | 4,50 | 0,63 | 3,58 | 0,41 | 4,34 | 0,22 | 4,33 | 0,07 | 7,12 |
| PSF i3s5 | 0,23 | 7,34 | 0,19 | 8,45 | 4,46 | 1,12 | 7,53 | 0,78 | 6,98 | 0,38 | 8,75 | 0,15 | 10,74 | 0,01 | 8,76 |
| PSF i5s5 | 0,17 | 9,84 | 0,13 | 10,42 | 4,55 | 1,37 | 7,35 | 1,10 | 7,36 | 0,53 | 11,36 | 0,25 | 11,64 | 0,02 | 8,44 |
| PSF i10s5 | 0,12 | 13,00 | 0,08 | 13,09 | 5,15 | 1,60 | 7,63 | 1,43 | 7,11 | 0,76 | 9,84 | 0,40 | 13,59 | 0,04 | 9,03 |
| PSF i15s5 | 0,10 | 13,78 | 0,06 | 14,58 | 5,57 | 1,66 | 8,07 | 1,54 | 7,07 | 0,85 | 9,28 | 0,46 | 13,16 | 0,06 | 11,66 |
| PSF i20s5 | 0,10 | 13,71 | 0,05 | 15,95 | 5,55 | 1,67 | 7,55 | 1,58 | 6,42 | 0,90 | 8,56 | 0,50 | 13,23 | 0,08 | 13,43 |
| PSF i25s5 | 0,10 | 13,39 | 0,05 | 16,35 | 5,73 | 1,66 | 7,32 | 1,59 | 6,21 | 0,93 | 7,97 | 0,51 | 13,30 | 0,09 | 14,20 |
| PSF i30s5 | 0,10 | 12,92 | 0,04 | 16,59 | 5,85 | 1,65 | 7,31 | 1,60 | 6,46 | 0,96 | 7,87 | 0,53 | 12,78 | 0,10 | 14,58 |
| PSF i20s5 Gauss 2mm | 0.10 | 14.97 | 0.06 | 17.56 | 3.47 | 1.10 | 3.85 | 0.92 | 3.12 | 0.56 | 4.86 | 0.28 | 8.59 | 0.06 | 10.85 |

Table S3. Image quality parameters calculated for the 18F-filled NEMA NU 4-2008 phantom in off-center position with 3 scattering sources placed in the FOV (OC-SC^+^). Results are presented for the different reconstruction settings tested.

| Recons | SOR_water_ | %SD water | SOR_air_ | % SD air | Uniformity %SD | RC 5mm | %SD 5mm | RC 4mm | %SD 4mm | RC 3mm | %SD 3mm | RC 2mm | %SD 2mm | RC 1mm | %SD 1mm |
| --- | --- | --- | --- | --- | --- | --- | --- | --- | --- | --- | --- | --- | --- | --- | --- |
| FBP | 0,17 | 18,57 | 0,08 | 44,06 | 3,79 | 0,56 | 4,30 | 0,45 | 5,63 | 0,30 | 7,73 | 0,14 | 9,32 | 0,03 | 28,43 |
| 3D-OSEM i3s5 | 0,24 | 11,19 | 0,20 | 12,61 | 2,21 | 0,67 | 2,36 | 0,58 | 3,24 | 0,35 | 2,36 | 0,18 | 5,18 | 0,03 | 9,02 |
| 3D-OSEM i5s5 | 0,20 | 11,89 | 0,15 | 13,71 | 2,58 | 0,67 | 3,29 | 0,59 | 3,72 | 0,37 | 2,83 | 0,22 | 5,38 | 0,04 | 8,76 |
| 3D-OSEM i10s5 | 0,17 | 13,14 | 0,12 | 15,53 | 3,15 | 0,66 | 3,75 | 0,57 | 4,32 | 0,38 | 3,85 | 0,22 | 6,04 | 0,06 | 10,25 |
| 3D-OSEM i15s5 | 0,17 | 14,08 | 0,11 | 16,88 | 3,53 | 0,66 | 4,05 | 0,57 | 4,70 | 0,37 | 4,61 | 0,22 | 6,58 | 0,06 | 11,67 |
| 3D-OSEM i20s5 | 0,16 | 14,83 | 0,10 | 18,03 | 3,81 | 0,67 | 4,30 | 0,57 | 4,98 | 0,37 | 5,15 | 0,22 | 6,97 | 0,06 | 12,68 |
| 3D-OSEM i25s5 | 0,10 | 25,12 | 0,10 | 19,09 | 4,02 | 0,67 | 4,49 | 0,57 | 5,20 | 0,37 | 5,54 | 0,21 | 7,26 | 0,06 | 13,48 |
| 3D-OSEM i30s5 | 0,10 | 25,99 | 0,01 | 200,57 | 4,19 | 0,67 | 4,64 | 0,57 | 5,38 | 0,37 | 5,83 | 0,21 | 7,48 | 0,06 | 14,15 |
| PSF i3s5 | 0,25 | 6,62 | 0,21 | 7,24 | 3,80 | 0,97 | 6,43 | 0,67 | 5,93 | 0,34 | 6,67 | 0,13 | 9,85 | 0,01 | 9,51 |
| PSF i5s5 | 0,19 | 8,61 | 0,15 | 9,55 | 3,76 | 1,20 | 6,52 | 0,93 | 6,02 | 0,48 | 8,20 | 0,22 | 6,67 | 0,02 | 9,22 |
| PSF i10s5 | 0,13 | 11,78 | 0,09 | 12,84 | 4,61 | 1,35 | 7,31 | 1,29 | 6,11 | 0,67 | 7,70 | 0,38 | 9,59 | 0,04 | 10,61 |
| PSF i15s5 | 0,10 | 14,02 | 0,07 | 15,51 | 5,25 | 1,41 | 9,76 | 1,44 | 7,36 | 0,76 | 8,23 | 0,42 | 9,92 | 0,06 | 10,30 |
| PSF i20s5 | 0,10 | 14,62 | 0,06 | 17,54 | 4,52 | 1,43 | 9,86 | 1,53 | 7,23 | 0,87 | 9,30 | 0,51 | 11,30 | 0,08 | 10,19 |
| PSF i25s5 | 0,09 | 15,00 | 0,05 | 18,65 | 4,73 | 1,43 | 9,93 | 1,54 | 7,43 | 0,92 | 10,09 | 0,56 | 13,55 | 0,08 | 10,28 |
| PSF i30s5 | 0,09 | 15,18 | 0,05 | 19,43 | 4,91 | 1,42 | 9,85 | 1,58 | 7,65 | 0,95 | 10,41 | 0,59 | 14,99 | 0,10 | 11,57 |

Table S4. Image quality parameters calculated for the 68Ga-filled NEMA NU 4-2008 phantom positioned in the center of the FOV (C-SC^-^). Results are presented for the different reconstruction settings tested.

| Recons | SOR_water_ | %SD water | SOR_air_ | % SD air | Uniformity %SD | RC 5mm | %SD 5mm | RC 4mm | %SD 4mm | RC 3mm | %SD 3mm | RC 2mm | %SD 2mm | RC 1mm | %SD 1mm |
| --- | --- | --- | --- | --- | --- | --- | --- | --- | --- | --- | --- | --- | --- | --- | --- |
| FBP | 0,19 | 18,56 | 0,14 | 18,52 | 3,43 | 0,44 | 3,97 | 0,33 | 3,78 | 0,20 | 5,33 | 0,10 | 6,84 | - | - |
| 3D-OSEM i3s5 | 0,24 | 12,24 | 0,21 | 11,10 | 2,98 | 0,49 | 3,49 | 0,38 | 3,03 | 0,23 | 3,94 | 0,10 | 5,43 | - | - |
| 3D-OSEM i5s5 | 0,21 | 13,23 | 0,17 | 11,80 | 3,14 | 0,48 | 3,85 | 0,38 | 3,25 | 0,24 | 4,67 | 0,12 | 6,45 | - | - |
| 3D-OSEM i10s5 | 0,18 | 14,76 | 0,14 | 13,35 | 3,45 | 0,48 | 4,33 | 0,38 | 3,59 | 0,24 | 5,81 | 0,12 | 8,02 | - | - |
| 3D-OSEM i15s5 | 0,17 | 16,69 | 0,12 | 14,00 | 3,67 | 0,47 | 4,63 | 0,38 | 3,83 | 0,24 | 6,59 | 0,12 | 8,86 | - | - |
| 3D-OSEM i20s5 | 0,17 | 17,33 | 0,12 | 14,77 | 3,84 | 0,47 | 4,85 | 0,38 | 4,04 | 0,24 | 7,14 | 0,12 | 9,60 | - | - |
| 3D-OSEM i25s5 | 0,17 | 17,80 | 0,12 | 15,46 | 3,98 | 0,47 | 5,02 | 0,38 | 4,21 | 0,24 | 7,55 | 0,12 | 10,25 | - | - |
| 3D-OSEM i30s5 | 0,16 | 18,18 | 0,11 | 16,08 | 4,10 | 0,47 | 5,16 | 0,38 | 4,37 | 0,24 | 7,84 | 0,12 | 10,82 | - | - |
| PSF i3s5 | 0,24 | 10,04 | 0,21 | 8,08 | 1,51 | 0,62 | 2,36 | 0,39 | 3,17 | 0,23 | 5,38 | 0,09 | 6,53 | - | - |
| PSF i5s5 | 0,19 | 11,22 | 0,16 | 9,65 | 1,53 | 0,70 | 2,60 | 0,48 | 2,36 | 0,30 | 2,90 | 0,13 | 5,31 | - | - |
| PSF i10s5 | 0,14 | 13,40 | 0,11 | 13,03 | 2,28 | 0,75 | 3,77 | 0,58 | 2,99 | 0,38 | 2,46 | 0,18 | 4,44 | - | - |
| PSF i15s5 | 0,12 | 14,76 | 0,09 | 15,34 | 2,63 | 0,75 | 4,05 | 0,59 | 3,56 | 0,41 | 3,24 | 0,20 | 5,12 | - | - |
| PSF i20s5 | 0,12 | 15,67 | 0,08 | 17,01 | 2,81 | 0,74 | 4,14 | 0,59 | 3,73 | 0,42 | 3,92 | 0,22 | 6,07 | - | - |
| PSF i25s5 | 0,11 | 16,30 | 0,07 | 18,28 | 2,94 | 0,73 | 4,16 | 0,58 | 3,78 | 0,43 | 4,28 | 0,22 | 6,91 | - | - |
| PSF i30s5 | 0,11 | 15,97 | 0,07 | 20,05 | 3,04 | 0,73 | 4,16 | 0,58 | 3,83 | 0,44 | 4,50 | 0,23 | 7,66 | - | - |

Table S5. Image quality parameters calculated for the 68Ga-filled NEMA NU 4-2008 phantom in off-center position with 3 scattering sources placed in the FOV (OC-SC^+^). Results are presented for the different reconstruction settings tested.

| Recons | SOR_water_ | %SD water | SOR_air_ | % SD air | Uniformity %SD | RC 5mm | %SD 5mm | RC 4mm | %SD 4mm | RC 3mm | %SD 3mm | RC 2mm | %SD 2mm | RC 1mm | %SD 1mm |
| --- | --- | --- | --- | --- | --- | --- | --- | --- | --- | --- | --- | --- | --- | --- | --- |
| FBP | 0,20 | 19,15 | 0,14 | 27,01 | 5,90 | 0,31 | 3,78 | 0,25 | 6,15 | 0,16 | 9,82 | 0,08 | 20,56 | - | - |
| 3D-OSEM i3s5 | 0,24 | 11,16 | 0,21 | 9,98 | 3,50 | 0,45 | 4,11 | 0,33 | 3,76 | 0,21 | 4,35 | 0,09 | 8,07 | - | - |
| 3D-OSEM i5s5 | 0,22 | 12,58 | 0,18 | 10,65 | 3,93 | 0,48 | 4,71 | 0,36 | 4,26 | 0,24 | 4,93 | 0,11 | 7,93 | - | - |
| 3D-OSEM i10s5 | 0,19 | 15,22 | 0,15 | 12,18 | 4,33 | 0,48 | 5,39 | 0,36 | 4,77 | 0,24 | 5,46 | 0,12 | 8,80 | - | - |
| 3D-OSEM i15s5 | 0,18 | 17,08 | 0,14 | 13,45 | 4,61 | 0,48 | 5,77 | 0,36 | 5,14 | 0,24 | 5,59 | 0,12 | 9,32 | - | - |
| 3D-OSEM i20s5 | 0,18 | 18,49 | 0,13 | 14,46 | 4,84 | 0,48 | 6,03 | 0,36 | 5,43 | 0,24 | 5,65 | 0,12 | 9,66 | - | - |
| 3D-OSEM i25s5 | 0,18 | 19,61 | 0,13 | 15,32 | 5,02 | 0,48 | 6,22 | 0,36 | 5,65 | 0,24 | 5,70 | 0,12 | 9,88 | - | - |
| 3D-OSEM i30s5 | 0,18 | 20,53 | 0,13 | 16,05 | 5,16 | 0,47 | 6,37 | 0,36 | 5,83 | 0,24 | 5,76 | 0,12 | 10,03 | - | - |
| PSF i3s5 | 0,26 | 7,85 | 0,23 | 7,39 | 1,79 | 0,62 | 3,76 | 0,38 | 3,54 | 0,21 | 5,37 | 0,07 | 5,58 | - | - |
| PSF i5s5 | 0,20 | 9,52 | 0,17 | 9,25 | 1,77 | 0,72 | 3,21 | 0,47 | 3,04 | 0,27 | 4,50 | 0,11 | 5,80 | - | - |
| PSF i10s5 | 0,15 | 12,24 | 0,12 | 12,35 | 2,48 | 0,77 | 3,38 | 0,55 | 3,45 | 0,36 | 4,40 | 0,16 | 5,33 | - | - |
| PSF i15s5 | 0,13 | 13,84 | 0,10 | 14,32 | 2,87 | 0,77 | 3,83 | 0,56 | 3,76 | 0,40 | 4,21 | 0,19 | 5,24 | - | - |
| PSF i20s5 | 0,12 | 14,95 | 0,08 | 15,70 | 2,62 | 0,76 | 3,88 | 0,56 | 3,67 | 0,41 | 3,92 | 0,20 | 4,77 | - | - |
| PSF i25s5 | 0,12 | 15,79 | 0,08 | 16,75 | 2,76 | 0,75 | 4,23 | 0,56 | 3,90 | 0,42 | 4,10 | 0,21 | 4,46 | - | - |
| PSF i30s5 | 0,11 | 16,47 | 0,07 | 17,57 | 2,90 | 0,75 | 4,59 | 0,56 | 4,15 | 0,43 | 4,39 | 0,22 | 4,20 | - | - |

Table S6. RC max and RC A50 calculated for the 18F Micro Hollow Sphere phantom with a contrast ratio of 1/8 for C-SC^-^ and OC-SC^+^ acquisitions.

|  | C-SC^-^ | | | | | | | | OC-SC^+^ | | | | | | | |
| --- | --- | --- | --- | --- | --- | --- | --- | --- | --- | --- | --- | --- | --- | --- | --- | --- |
|  | RC max | | | | RC A50 | | | | RC max | | | | RC A50 | | | |
| Φ spheres (mm) | 7.86 | 6.23 | 4.95 | 3.95 | 7.86 | 6.23 | 4.95 | 3.95 | 7.86 | 6.23 | 4.95 | 3.95 | 7.86 | 6.23 | 4.95 | 3.95 |
| 3D-OSEM i3s5 | 0,94 | 0,77 | 0,55 | 0,37 | 0,70 | 0,58 | 0,42 | 0,30 | 0,91 | 0,75 | 0,55 | 0,32 | 0,69 | 0,54 | 0,41 | 0,26 |
| 3D-OSEM i5s5 | 0,91 | 0,80 | 0,61 | 0,43 | 0,69 | 0,60 | 0,46 | 0,34 | 0,90 | 0,77 | 0,60 | 0,35 | 0,67 | 0,56 | 0,44 | 0,29 |
| 3D-OSEM i10s5 | 0,88 | 0,78 | 0,63 | 0,47 | 0,68 | 0,59 | 0,47 | 0,37 | 0,88 | 0,76 | 0,61 | 0,37 | 0,66 | 0,55 | 0,44 | 0,30 |
| 3D-OSEM i15s5 | 0,87 | 0,77 | 0,63 | 0,48 | 0,68 | 0,59 | 0,47 | 0,37 | 0,87 | 0,76 | 0,60 | 0,38 | 0,66 | 0,55 | 0,44 | 0,30 |
| 3D-OSEM i20s5 | 0,87 | 0,77 | 0,63 | 0,48 | 0,68 | 0,58 | 0,47 | 0,37 | 0,87 | 0,76 | 0,60 | 0,38 | 0,66 | 0,55 | 0,44 | 0,30 |
| 3D-OSEM i25s5 | 0,88 | 0,77 | 0,63 | 0,48 | 0,68 | 0,58 | 0,47 | 0,37 | 0,87 | 0,76 | 0,60 | 0,37 | 0,66 | 0,55 | 0,44 | 0,30 |
| 3D-OSEM i30s5 | 0,88 | 0,77 | 0,63 | 0,48 | 0,68 | 0,58 | 0,47 | 0,37 | 0,87 | 0,76 | 0,60 | 0,37 | 0,66 | 0,55 | 0,44 | 0,30 |
| PSF i3s5 | 1,43 | 0,89 | 0,49 | 0,29 | 0,99 | 0,65 | 0,38 | 0,23 | 1,30 | 0,85 | 0,48 | 0,26 | 0,91 | 0,60 | 0,36 | 0,22 |
| PSF i5s5 | 1,72 | 1,16 | 0,66 | 0,36 | 1,17 | 0,84 | 0,49 | 0,29 | 1,55 | 1,14 | 0,64 | 0,32 | 1,08 | 0,80 | 0,47 | 0,26 |
| PSF i10s5 | 1,90 | 1,52 | 0,94 | 0,52 | 1,29 | 1,08 | 0,69 | 0,41 | 1,72 | 1,53 | 0,93 | 0,41 | 1,20 | 1,09 | 0,67 | 0,36 |
| PSF i15s5 | 1,86 | 1,68 | 1,13 | 0,63 | 1,28 | 1,19 | 0,82 | 0,50 | 1,72 | 1,73 | 1,13 | 0,52 | 1,21 | 1,21 | 0,80 | 0,42 |
| PSF i20s5 | 1,79 | 1,77 | 1,25 | 0,72 | 1,25 | 1,26 | 0,90 | 0,56 | 1,68 | 1,84 | 1,28 | 0,58 | 1,19 | 1,29 | 0,90 | 0,47 |
| PSF i25s5 | 1,71 | 1,82 | 1,35 | 0,80 | 1,20 | 1,30 | 0,96 | 0,61 | 1,63 | 1,90 | 1,38 | 0,63 | 1,16 | 1,34 | 1,00 | 0,51 |
| PSF i30s5 | 1,63 | 1,85 | 1,42 | 0,86 | 1,17 | 1,33 | 1,04 | 0,66 | 1,58 | 1,95 | 1,47 | 0,68 | 1,13 | 1,37 | 1,07 | 0,54 |
| PSF i20s5 Gauss 2mm | 1,43 | 1,27 | 0,84 | 0,51 | 1,02 | 0,92 | 0,62 | 0,40 | 1,40 | 1,22 | 0,80 | 0,44 | 0,98 | 0,88 | 0,59 | 0,36 |

Table S7. RC max and RC A50 calculated for the 18F Micro Hollow Sphere phantom with a contrast ratio of 1/4 for C-SC^-^ and OC-SC^+^ acquisitions.

|  | C-SC^-^ | | | | | | | | OC-SC^+^ | | | | | | | |
| --- | --- | --- | --- | --- | --- | --- | --- | --- | --- | --- | --- | --- | --- | --- | --- | --- |
|  | RC Amax | | | | RC A50 | | | | RC Amax | | | | RC A50 | | | |
| Φ spheres (mm) | 7.86 | 6.23 | 4.95 | 3.95 | 7.86 | 6.23 | 4.95 | 3.95 | 7.86 | 6.23 | 4.95 | 3.95 | 7.86 | 6.23 | 4.95 | 3.95 |
| 3D-OSEM i3s5 | 0,91 | 0,72 | 0,50 | 0,37 | 0,70 | 0,57 | 0,38 | 0,33 | 0,89 | 0,71 | 0,47 | 0,32 | 0,68 | 0,55 | 0,39 | 0,29 |
| 3D-OSEM i5s5 | 0,91 | 0,77 | 0,56 | 0,42 | 0,70 | 0,61 | 0,46 | 0,36 | 0,90 | 0,76 | 0,52 | 0,34 | 0,69 | 0,57 | 0,43 | 0,31 |
| 3D-OSEM i10s5 | 0,92 | 0,77 | 0,61 | 0,47 | 0,72 | 0,61 | 0,49 | 0,40 | 0,88 | 0,76 | 0,55 | 0,37 | 0,68 | 0,58 | 0,44 | 0,33 |
| 3D-OSEM i15s5 | 0,92 | 0,76 | 0,62 | 0,49 | 0,72 | 0,60 | 0,50 | 0,41 | 0,88 | 0,75 | 0,55 | 0,38 | 0,68 | 0,58 | 0,45 | 0,34 |
| 3D-OSEM i20s5 | 0,92 | 0,75 | 0,62 | 0,50 | 0,72 | 0,60 | 0,50 | 0,41 | 0,88 | 0,74 | 0,55 | 0,38 | 0,68 | 0,58 | 0,45 | 0,34 |
| 3D-OSEM i25s5 | 0,93 | 0,75 | 0,62 | 0,50 | 0,71 | 0,60 | 0,50 | 0,42 | 0,89 | 0,74 | 0,55 | 0,38 | 0,68 | 0,58 | 0,45 | 0,34 |
| 3D-OSEM i30s5 | 0,92 | 0,75 | 0,62 | 0,50 | 0,72 | 0,60 | 0,50 | 0,42 | 0,89 | 0,74 | 0,55 | 0,38 | 0,68 | 0,58 | 0,45 | 0,34 |
| PSF i3s5 | 1,08 | 0,70 | 0,42 | 0,30 | 0,83 | 0,55 | 0,36 | 0,28 | 1,07 | 0,67 | 0,41 | 0,28 | 0,78 | 0,52 | 0,35 | 0,26 |
| PSF i5s5 | 1,32 | 0,90 | 0,51 | 0,34 | 1,01 | 0,69 | 0,42 | 0,31 | 1,31 | 0,86 | 0,48 | 0,30 | 0,93 | 0,65 | 0,40 | 0,28 |
| PSF i10s5 | 1,57 | 1,22 | 0,67 | 0,42 | 1,18 | 0,91 | 0,53 | 0,36 | 1,58 | 1,18 | 0,63 | 0,35 | 1,11 | 0,87 | 0,50 | 0,32 |
| PSF i15s5 | 1,64 | 1,40 | 0,78 | 0,49 | 1,24 | 1,02 | 0,59 | 0,41 | 1,67 | 1,02 | 0,59 | 0,41 | 1,16 | 0,99 | 0,57 | 0,34 |
| PSF i20s5 | 1,65 | 1,52 | 0,86 | 0,54 | 1,24 | 1,11 | 0,67 | 0,45 | 1,69 | 1,51 | 0,82 | 0,42 | 1,18 | 1,08 | 0,63 | 0,36 |
| PSF i25s5 | 1,65 | 1,60 | 0,92 | 0,58 | 1,25 | 1,16 | 0,71 | 0,48 | 1,68 | 1,60 | 0,89 | 0,45 | 1,18 | 1,16 | 0,68 | 0,38 |
| PSF i30s5 | 1,64 | 1,65 | 0,96 | 0,62 | 1,23 | 1,20 | 0,74 | 0,51 | 1,66 | 1,68 | 0,94 | 0,47 | 1,17 | 1,22 | 0,71 | 0,40 |
| PSF i20s5 Gauss 2mm | 1,34 | 1,09 | 0,65 | 0,42 | 1,01 | 0,83 | 0,53 | 0,38 | 1,32 | 1,06 | 0,62 | 0,37 | 0,96 | 0,80 | 0,50 | 0,33 |

Table S8. RC max and RC A50 calculated for the 68Ga Micro Hollow Sphere phantom with a contrast ratio of 1/8 for C-SC^-^ and OC-SC^+^ acquisitions

|  | C-SC^-^ | | | | | | | | OC-SC^+^ | | | | | | | |
| --- | --- | --- | --- | --- | --- | --- | --- | --- | --- | --- | --- | --- | --- | --- | --- | --- |
|  | RC max | | | | RC A50 | | | | RC max | | | | RC A50 | | | |
| Φ spheres (mm) | 7.86 | 6.23 | 4.95 | 3.95 | 7.86 | 6.23 | 4.95 | 3.95 | 7.86 | 6.23 | 4.95 | 3.95 | 7.86 | 6.23 | 4.95 | 3.95 |
| 3D-OSEM i3s5 | 0,63 | 0,41 | 0,29 | 0,19 | 0,46 | 0,31 | 0,22 | 0,16 | 0,59 | 0,40 | 0,27 | 0,17 | 0,43 | 0,30 | 0,21 | 0,15 |
| 3D-OSEM i5s5 | 0,63 | 0,42 | 0,31 | 0,21 | 0,45 | 0,31 | 0,24 | 0,17 | 0,59 | 0,41 | 0,28 | 0,18 | 0,43 | 0,31 | 0,22 | 0,16 |
| 3D-OSEM i10s5 | 0,62 | 0,42 | 0,32 | 0,23 | 0,46 | 0,32 | 0,25 | 0,18 | 0,59 | 0,40 | 0,29 | 0,19 | 0,43 | 0,31 | 0,23 | 0,16 |
| 3D-OSEM i15s5 | 0,62 | 0,41 | 0,32 | 0,23 | 0,46 | 0,32 | 0,25 | 0,19 | 0,59 | 0,40 | 0,29 | 0,20 | 0,43 | 0,31 | 0,23 | 0,17 |
| 3D-OSEM i20s5 | 0,62 | 0,41 | 0,32 | 0,24 | 0,46 | 0,32 | 0,25 | 0,19 | 0,59 | 0,39 | 0,30 | 0,20 | 0,43 | 0,31 | 0,23 | 0,17 |
| 3D-OSEM i25s5 | 0,62 | 0,41 | 0,32 | 0,24 | 0,46 | 0,32 | 0,25 | 0,19 | 0,59 | 0,39 | 0,30 | 0,20 | 0,43 | 0,31 | 0,23 | 0,17 |
| 3D-OSEM i30s5 | 0,62 | 0,41 | 0,32 | 0,24 | 0,46 | 0,32 | 0,25 | 0,19 | 0,59 | 0,39 | 0,30 | 0,20 | 0,43 | 0,31 | 0,23 | 0,17 |
| PSF i3s5 | 0,82 | 0,48 | 0,28 | 0,17 | 0,58 | 0,35 | 0,22 | 0,15 | 0,75 | 0,45 | 0,27 | 0,16 | 0,54 | 0,33 | 0,21 | 0,14 |
| PSF i5s5 | 0,92 | 0,57 | 0,34 | 0,20 | 0,64 | 0,41 | 0,26 | 0,16 | 0,84 | 0,55 | 0,31 | 0,18 | 0,60 | 0,39 | 0,24 | 0,15 |
| PSF i10s5 | 0,96 | 0,68 | 0,43 | 0,24 | 0,67 | 0,47 | 0,31 | 0,19 | 0,89 | 0,65 | 0,38 | 0,21 | 0,63 | 0,45 | 0,28 | 0,18 |
| PSF i15s5 | 0,94 | 0,71 | 0,47 | 0,26 | 0,67 | 0,50 | 0,35 | 0,21 | 0,87 | 0,69 | 0,42 | 0,23 | 0,63 | 0,48 | 0,31 | 0,19 |
| PSF i20s5 | 0,92 | 0,73 | 0,50 | 0,28 | 0,66 | 0,51 | 0,37 | 0,22 | 0,86 | 0,70 | 0,44 | 0,25 | 0,62 | 0,49 | 0,32 | 0,20 |
| PSF i25s5 | 0,90 | 0,74 | 0,53 | 0,29 | 0,65 | 0,51 | 0,38 | 0,23 | 0,85 | 0,71 | 0,45 | 0,26 | 0,61 | 0,50 | 0,33 | 0,21 |
| PSF i30s5 | 0,88 | 0,74 | 0,54 | 0,30 | 0,64 | 0,52 | 0,39 | 0,24 | 0,84 | 0,72 | 0,46 | 0,27 | 0,60 | 0,50 | 0,34 | 0,21 |
