# Supplementary material for: PET imaging and quantification of small animals using a clinical SiPM-based camera
Source: EJNMMI Phys. 2023 Oct 7;10:61. doi: 10.1186/s40658-023-00583-2 (PMC10560240; doi:10.1186/s40658-023-00583-2)
Supplement: Supplementary file 2 — Additional file 2. SPECT images of the NEMA N4-2008 phantom and CT images of the 3D-printed rat phantoms. [file 40658_2023_583_MOESM2_ESM.docx]

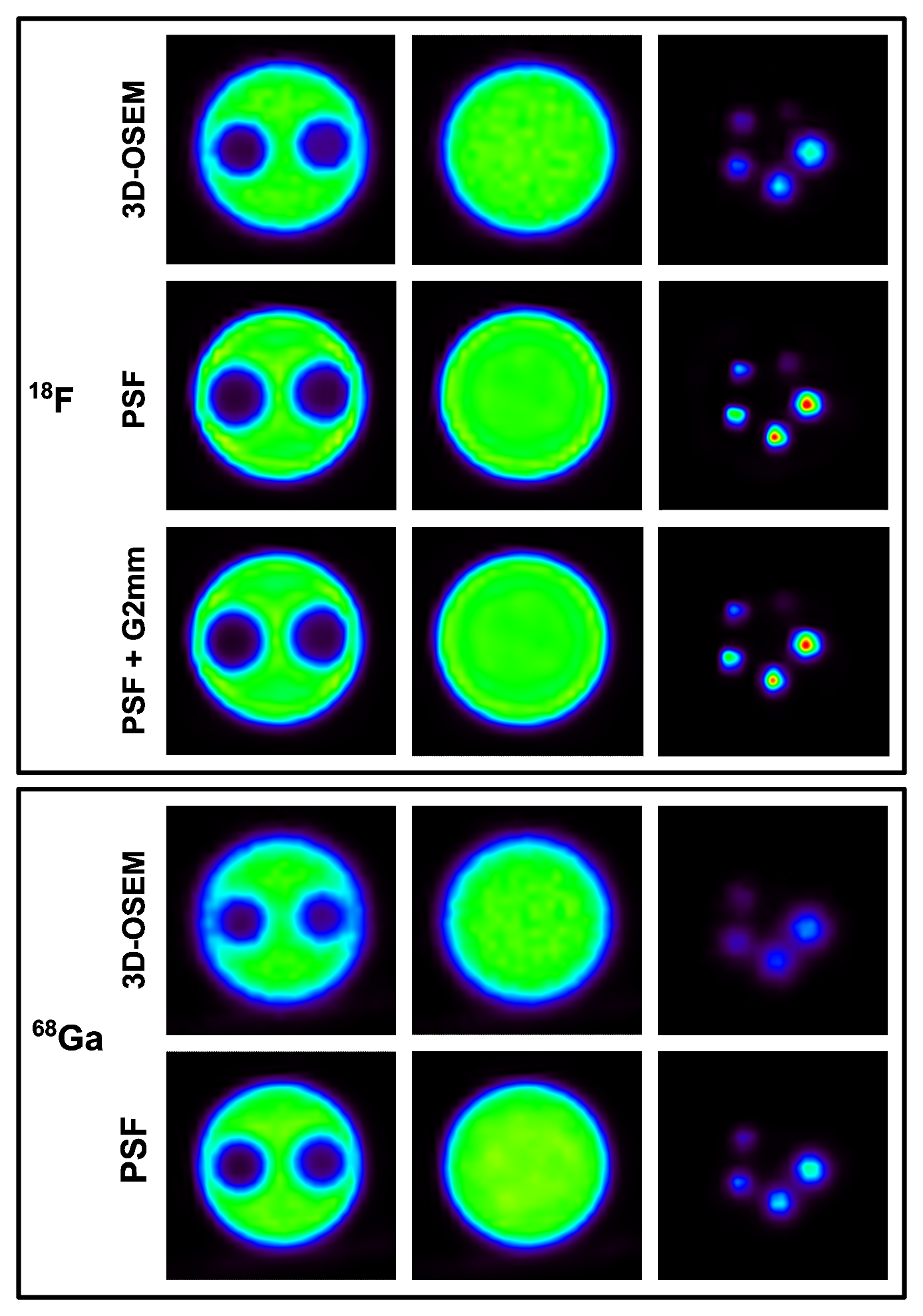


Figure S1: Image samples of 18F and 68Ga-filled NEMA NU 4-2008 phantoms performed for C-SC^-^ acquisitions. Three different reconstruction settings are presented: 3D-OSEM, PSF and PSF with a 2 mm FWHM Gaussian filter only for 18F acquisition. Axial slices are centred in the three different parts of the phantom: air and water inserts (left), uniform region (middle) and capillaries (right). The image window was normalized on the background of the phantom.


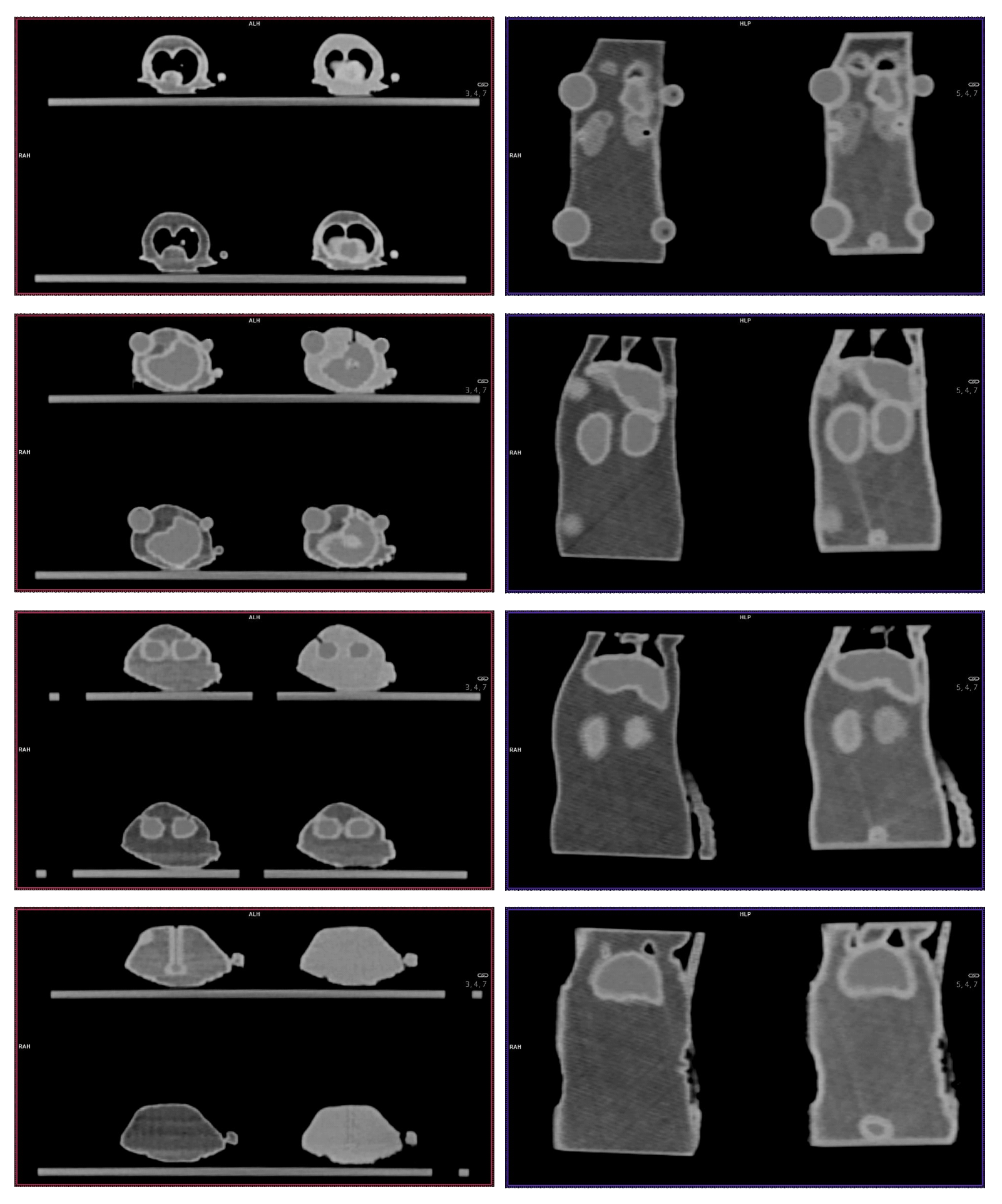


Figure S2: CT images of the four 3D-printed rat phantoms filled with water. Images were reconstructed with a soft tissue kernel filter and displayed with a window width of 500 Hounsfield unit (HU) and a window level of +50 HU. Four axial (left) and coronal (right) slices are presented, showing the different compartments of the phantom.
